# CIHR Canadian HIV Trials Network Coinfection and Concurrent Diseases Core Research Group: 2016 Updated Canadian HIV/Hepatitis C Adult Guidelines for Management and Treatment

**DOI:** 10.1155/2016/4385643

**Published:** 2016-07-04

**Authors:** Mark Hull, Stephen Shafran, Alex Wong, Alice Tseng, Pierre Giguère, Lisa Barrett, Shariq Haider, Brian Conway, Marina Klein, Curtis Cooper

**Affiliations:** ^1^British Columbia Centre for Excellence in HIV/AIDS, University of British Columbia, Vancouver, BC, Canada V6T 1Z4; ^2^University of Alberta, Edmonton, AB, Canada T6G 2R3; ^3^Regina Qu'Appelle Health Region, Regina, SK, Canada S4P 1E2; ^4^Toronto General Hospital, Toronto, ON, Canada M5G 2C4; ^5^The Ottawa Hospital, Ottawa, ON, Canada K1H 8L6; ^6^Dalhousie University, Halifax, NS, Canada B3H 4R2; ^7^McMaster University, Hamilton, ON, Canada L8S 4L8; ^8^Vancouver Infectious Diseases Centre, Vancouver, BC, Canada V6Z 2C7; ^9^McGill University, Montreal, QC, Canada H3A 0G4; ^10^The Ottawa Hospital, General Campus, G12, 501 Smyth Road, Ottawa, ON, Canada K1H 8L6

## Abstract

*Background*. Hepatitis C virus (HCV) coinfection occurs in 20–30% of Canadians living with HIV and is responsible for a heavy burden of morbidity and mortality.* Purpose*. To update national standards for management of HCV-HIV coinfected adults in the Canadian context with evolving evidence for and accessibility of effective and tolerable DAA therapies. The document addresses patient workup and treatment preparation, antiviral recommendations overall and in specific populations, and drug-drug interactions.* Methods*. A standing working group with HIV-HCV expertise was convened by The Canadian Institute of Health Research HIV Trials Network to review recently published HCV antiviral data and update Canadian HIV-HCV Coinfection Guidelines.* Results*. The gap in sustained virologic response between HCV monoinfection and HIV-HCV coinfection has been eliminated with newer HCV antiviral regimens. All coinfected individuals should be assessed for interferon-free, Direct Acting Antiviral HCV therapy. Regimens vary in content, duration, and success based largely on genotype. Reimbursement restrictions forcing the use of pegylated interferon is not acceptable if optimal patient care is to be provided.* Discussion*. Recommendations may not supersede individual clinical judgement. Treatment advances published since December 2015 are not considered in this document.

## 1. Introduction

Continued improvements in combination antiretroviral therapy (ART) have resulted in sustained gains in projected life expectancy for HIV-infected individuals [1, 2]. Long-term management of HIV now increasingly requires assessment and appropriate interventions for comorbid conditions that may impact long-term morbidity and mortality to a greater extent than HIV infection itself. Mortality secondary to chronic hepatitis C virus (HCV) infection has now surpassed that of HIV in the United States [3] in general and is a cause of significant mortality in coinfected individuals in the ART era [4, 5]. Management of HIV-HCV coinfected individuals is more complex relative to HIV or HCV monoinfected patients, with issues related to accelerated progression of liver disease, timing and nature of ART and HCV therapy, addictions management, and the advent of direct acting antiviral agents for HCV therapy, potential for clinically significant drug-drug interactions with ART regimens. In order to develop national standards for the management of coinfected individuals, The Canadian Institute of Health Research HIV Trials Network convened a standing panel of physicians and pharmacists with specific expertise in HIV-HCV coinfection tasked with reviewing the current literature, existing guidelines and protocols and adapting them to the Canadian context. This document reflects the consensus recommendations of this panel and was approved by the committee at large. In an effort to characterize the quality of evidence supporting these recommendations, a class (reflecting benefit verses harm) and level (assessing strength of certainty) of evidence were utilized. This system was used for Canadian Association for the Study of the Liver Guidelines for HCV and HBV management [6, 7] (Table 1). These recommendations are intended to aid clinicians in the management of the coinfected patient but may not supersede individual clinical judgement. Given the rapid advances in DAA-based HCV antiviral therapy an updated HIV-HCV guidelines document is required at this time.

## 2. Epidemiology of HIV and Hepatitis C Virus Coinfection

### 2.1. Epidemiology

In the three decades since HIV was identified, tremendous progress has been made in its treatment. Once universally fatal, characterized by AIDS-related opportunistic infections and malignancies, HIV infection now has been rendered a manageable chronic condition in developed countries through effective combination therapies [8]. HIV-infected individuals are now surviving for decades after acquiring their infection [9], resulting in comorbidities such as HCV coinfection now emerging as significant health problems for HIV-infected persons. Indeed, end- stage liver disease (ESLD) due to HCV is now a primary cause of morbidity and mortality in HIV-infected persons [10], including Canada [4].

HCV infection is recognized as one of the fastest growing health problems facing both developed industrialised countries and developing regions with an estimated 170 million persons [11, 12] and 220,000 Canadians chronically infected (0.64–0.71% of the population) and 44% of these remain undiagnosed [13]. In 2008, 13,127 persons (20% of the HIV-infected population) were estimated to be coinfected in Canada, with significant geographic variations [14]. Currently, injection drug use (IDU) is the main mode of HCV transmission (responsible for about 80% of infections) and is an important risk for HIV infection accounting for an estimated 14% of new HIV infections in 2011 [15]. Although the proportion of new HIV infections attributable to IDU has been in decline over the last decade amongst men; an increasing trend among women has been observed since 2003. In sentinel sites in the provinces of Quebec and Ontario, HIV seroprevalence amongst people who inject drugs (PWID) peaked at 18.6% in 2003. From 2003 to June 2008, their prevalence of HCV infection was 63%, and the overall proportion of those coinfected with HIV and HCV was 13% [16]. Receipt of contaminated blood products was once a key risk factor for HIV and HCV exposure [17]. However, the risk in Canada has been negligible for several decades [18].

Aboriginals, women, and youth who injected drugs are at particular risk for HIV and HCV infection [14, 19–22]. Aboriginal people comprised 3.8% of the Canadian population but 8.9% of prevalent HIV infections in 2011 [15]. HIV diagnosis amongst Aboriginal women was 14 times more common than amongst non-Aboriginal women in 1999–2003, the gap increasing to almost 20 times the nonindigenous rate in 2004–2008 [23]. High rates of IDU are resulting in parallel increases in HCV coinfection. The highest rates of these new HIV diagnoses are in Saskatchewan; 75% of which are associated with IDU and consequently HCV coinfection rates approach 90% [24]. The vulnerability of Aboriginals is illustrated with data from the Canadian Coinfection Cohort study (CTN222), a CIHR-funded prospective cohort that follows 1370 persons with HIV-HCV coinfection (http://www.cocostudy.ca/) [25]. Aboriginal peoples are disproportionately represented in the cohort: 16% of the cohort overall and 33% in British Columbia self-identified as Aboriginal and a very high proportion of these were women (62%). Overall, 458 (57%) had been previously incarcerated (78% of Aboriginal peoples versus 53% of non-Aboriginal peoples). There are very high rates of past and current (past 6 months) substance use amongst participants with 81% reporting a history of IDU (38% were currently injecting; 23% sharing needles) [25].

Another important population at risk is persons incarcerated in correctional facilities. The elevated prevalence of HIV and HCV infections amongst inmates has been closely linked to IDU and the sharing of injection equipment. Reports have shown that 30%–50% of Canadian inmates have a history of IDU. Overall, 24% of Federal and 23% of provincial prisoners were HCV positive in 2011 [13]. In Ontario, HCV infections rates were 30% in female and 55% in male PWID which were remanded in provincial facilities. The prevalence of HCV-HIV coinfection was 1.2% among men and 1.5% amongst women. It was highest amongst older inmates and PWID [26]. In federal penitentiaries, 31% of those ever tested for HCV reported being positive. Aboriginal women reported the highest rate of 49%, more than 50% greater than the rates amongst non-Aboriginal women (30%) and all men (30.8%) [27]. The considerable movement between correctional populations both within and outside the correctional system presents numerous opportunities for HIV and HCV transmission.

Acquisition of HCV often occurs rapidly following initiation of injection drug use, either from the needle itself or the injecting paraphernalia [28, 29]. In addition, noninjection smoking paraphernalia has been implicated in HCV transmission [30].

Given the risk of HCV morbidity and the high costs of treating HCV amongst PWID (see economic impact below), evidence-based harm reduction strategies should be implemented for all populations at risk [30–34]. Given the high rate of ongoing drug use during periods of incarceration, often associated with elevated risks of needle sharing, harm reduction strategies for incarcerated individuals should also be considered [35–37].

Counselling regarding risk of acquiring HIV in HCV monoinfected individuals should be undertaken at time of original diagnosis, as subsequent HIV infection may occur if risk behaviours continue [38]. All HCV-infected individuals should undergo baseline HIV testing, with repeat testing recommended for those with ongoing risk behaviours for HIV transmission.

### 2.2. Sexual Transmission of HCV

Sexual transmission of HCV amongst heterosexuals is rare, estimated at 1 in 190,000 episodes of intercourse [39]. In contrast, acute HCV infection from sexual transmission has been increasingly observed in HIV+ men who have sex with men (MSM) [40–42], in whom HCV prevalence now ranges from 4 to 20% [41, 43]. In 2008 in Canada it was estimated that a total of 1316 MSM were HIV-HCV coinfected. In a recent systematic review, HIV+/MSM had rates of acute HCV that were 4.1 times higher (6.08 per 1000 person-years) than those of HIV−/MSM (1.48 per 1000 person-years), which is closer to that seen in the general population and clearly supports routine screening for this population [44]. The reasons why HIV-positive MSM might be at increased risk for acquiring HCV, an organism that is predominately associated with parenteral exposure, have not been fully determined. Nor is it clear whether HIV itself enhances either susceptibility or transmission of HCV. Unreported IDU, as well as serosorting, whereby unprotected anal sex occurs only with partners of the same HIV status as their own [45], may be contributory. Factors considered in the HIV+/MSM population include sexual practices that might lead to transmission through blood contact or concomitant sexually transmitted infections (genital ulcer disease) that might increase susceptibility of transmission rates [46, 47] and higher HCV viral loads in the context of HIV and HCV coinfection [47]. Methamphetamine and other recreational drug use have also been associated with HCV infection in HIV seropositive MSM [48]. Transmission networks appear to be quite important in most of the recent reports of acute HCV amongst HIV+/MSM, raising the possibility that certain viral strains may play a role [49]. The European AIDS Treatment Network (NEAT) Consensus panel on acute HCV in MSM has previously recommended consideration of screening MSM at risk for acute HCV with liver enzymes every 6 months and HCV antibody test annually. For those with ongoing IDU or recent sexually transmitted infection, screening every three months was recommended by the NEAT panel [50]. A recent cost-effectiveness analysis of screening options to detect acute HCV amongst MSM concluded that the strategy of liver enzymes every six months in combination with annual HCV screening was cost-effective in communities with incidence ≤1, 25 per 100 person-years, while screening with liver enzymes every 3 months was optimal in communities with higher incidence [51].

### 2.3. Distribution of HCV Genotype in Canada

Historically, the most important predictor of treatment response has been HCV genotype, with more favourable responses to pegylated interferon and ribavirin seen amongst genotypes 2 and 3 and lower response rates in genotypes 1 and 4. In Canada, 62% of HCV infections are of genotype 1. Amongst PWID genotypes 1 and 3 are most common. Genotype 2a is more frequent in patients previously exposed to multiple injections, surgery, or transfusions, and genotype 4 more common in African immigrants while genotype 6 is seen more commonly in immigrants from Asia. Some recent global outbreaks of HCV amongst HIV+ MSM have been attributed to genotype 4 infections [52]. The existence of all genotypes in Canada despite low prevalence of HCV reflects the diversity of the population, active immigration, and travel [53, 54].

### 2.4. The Effect of HIV on Natural History of HCV

In HCV monoinfection without concurrent excess alcohol consumption, a minimum of 20–30 years is typically required for HCV to cause significant liver disease such as cirrhosis or hepatocellular carcinoma (HCC) [55–59]. HIV-infected individuals, when exposed to HCV, are less likely to be spontaneously clear of infection [60, 61]. EuroSIDA reported that 23% of anti-HCV positive HIV-infected individuals tested HCV RNA negative [62]. Patients who are coinfected with HIV and HCV have more rapid fibrosis progression, higher rates of hepatocellular carcinoma and hepatic decompensation, and increased mortality compared to patients monoinfected with HCV [63, 64]. Successful treatment of HCV resulting in sustained virological response (SVR) in coinfected individuals reduces the incidence of liver-related events and improves all cause mortality [65].

A recent study of 435 liver biopsy pairs from a prospective cohort of 282 coinfected individuals without cirrhosis suggests that many have rapid and progressive fibrosis [66]. Fibrosis progression (defined as an increase of at least one METAVIR stage) was found in 97 of 282 patients (34%), with a median duration of 2.5 years between biopsy pairs. 39 biopsy pairs (9%) revealed progression of 2 or more METAVIR stages. Similar results have been reported previously [6]. Additional risk factors for progression of cirrhosis in coinfected individuals mirror that of monoinfected individuals and include alcohol intake, high body mass index, older age, and diabetes [63, 67–69].

In a large prospective cohort of 638 adults coinfected with HIV and HCV who received a baseline liver biopsy, hepatic fibrosis stage was independently associated with a composite outcome that included end-stage liver disease (ESLD), hepatocellular carcinoma, and death [70]. Compared with patients who had METAVIR stage 0 at baseline, patients with stage 2 at baseline had an incidence rate ratio of 2.31 (95% CI 1.23–4.34) while those with stage 4 at baseline had an incidence rate ratio of 3.57 (95% CI 2.06–6.19).

The rapid progression of liver fibrosis seen in coinfected individuals drives high rates of liver-related mortality observed worldwide in developed countries in the post-ART era. A Euro-SIDA analysis found that liver-related death accounted for 21.6% of deaths in a cohort of 3941 HCV antibody positive HIV patients [71]. Predictors of increased risk included presence of stage 2 or greater fibrosis at the time of initial assessment, low CD4 cell count, and hepatitis B surface antigen positivity. In the D:A:D cohort, 14.5% of all deaths recorded were secondary to liver-related causes (the most frequent cause of non-AIDS-related mortality), and 66% of these deaths occurred in coinfected individuals [5]. The proportion of deaths from ESLD in HIV-infected individuals in France increased from 2% in 1995 to 17% in 2005, attributable largely to coinfection with HCV [72]. In the Canadian Co-Infection Cohort, high rates of fibrosis progression and clinical ESLD events have been observed at rates six times higher than those reported in HCV monoinfected populations infected for a similar duration. ESLD has emerged as the primary cause of death amongst Canadian Co-Infection Cohort participants. These data may underestimate the true burden of disease in this population since the cohort only evaluates patients engaged in regular care.

Coinfected individuals are more likely to develop HCC at a young age in comparison to monoinfected individuals. In a retrospective study of 63 coinfected individuals with hepatocellular carcinoma, HIV-positive individuals were younger and developed HCC more quickly than HIV-negative controls [73]. The incidence of HCC in HIV-infected individuals continues to increase, as demonstrated in separate French and Spanish studies, due almost exclusively to coinfection with HCV [72, 74].

### 2.5. Health Economic Impact of HCV

In the Ontario Burden of Infectious Disease Study, HCV had the highest burden of disease as measured by years of life lost due to premature mortality and year-equivalents of reduced functioning, outranking all other infectious pathogens including HIV and* Streptococcus pneumoniae* [75]. In Canada, while the prevalence of HCV infection is predicted to decline over the next 20 years, rates of advanced liver disease and related complications will continue to rise over the same time period, and total healthcare expenditures secondary to HCV are predicted to increase by 60% from 2013 to a peak in 2032 [76], with the majority attributable to cirrhosis and its complications (81% in 2032 versus 56% in 2013). The lifetime cost per HCV-infected person in 2013 was estimated to be $64,694. HCV continues to remain the primary reason for liver transplantation in the developed world (Section 10).

*Recommendations*

1. All HIV+ persons should undergo screening for HCV antibodies when first evaluated. Screening should be repeated periodically at least annually, particularly for high risk individuals found to be initially negative (such as active injection drug users, Aboriginal peoples living in poverty or in marginalized circumstances, and persons who are/have been incarcerated) (Class 1, Level C).
2. HIV+ MSM should undergo screening for HCV antibodies annually in combination with liver enzymes every 6 months if sexually active with high risk behaviours, and repeat HCV antibody (with consideration of additional HCV RNA testing) should be performed whenever unexplained elevations in liver enzymes are noted (Class 2a, Level C).
3. Screening for HCV in HIV-infected individuals provides opportunities for prevention of transmission, risk-reduction, counselling, and linkage to care and harm reduction services (Class 1, Level C).

## 3. Managing HIV in the Setting of Coinfection

The management of HIV infection in coinfection requires consideration of a number of factors:

1. Effect of antiretroviral therapy on the natural history of liver disease.
2. Timing of initiation of antiretroviral therapy.
3. Risk of hepatotoxicity when antiretroviral therapy is initiated.
4. Potential for drug-drug interactions when undertaking HCV therapy.
5. Adherence to ART and HCV therapy, particularly in those with active addictions concerns.

### 3.1. Effects of Antiretroviral Therapy on HCV Natural History and Timing of Antiretroviral Initiation

Coinfected individuals experience faster progression of HCV disease, with higher risk of ESLD, particularly when both HIV and HCV remain untreated [77, 78]. In an analysis of coinfected individuals in the pre-ART era, the mean time period from infection to cirrhosis was as short as 6.9 years compared to 23.2 years in monoinfected patients [78]. Subsequent data have suggested that initiation of ART may serve to slow the rate of fibrosis progression and hence delay the onset of ESLD. Bräu et al. conducted a retrospective analysis of 656 HCV patients (274 HIV-infected) and determined a fibrosis progression rate as biopsy-determined fibrosis score/duration of HCV infection [79]. Fibrosis progression rates were highest in HIV-infected individuals with detectable HIV RNA but were similar in HIV-infected individuals with suppressed viral load and in HCV monoinfected patients. Another analysis has shown protective effect of longer duration of ART therapy and reduced biopsy-proven fibrosis [80].

Initiation of ART has also been shown to reduce liver-related mortality in coinfected patients. In a cohort of 285 coinfected patients initiating either limited antiretroviral therapy (*n* = 55) or full ART (*n* = 93) or remaining untreated between 1990 and 2002, liver-related mortality rates were lowest in those receiving ART (0.45 per 100 person-years) or dual therapy (0.69 per 100 person-years) and highest in those who received no therapy (1.70 per 100 person-years) [81]. In a cohort of 472 HIV-infected patients (256 coinfected with HCV), 41% of overall mortality was due to liver-related deaths and in Cox regression analysis, receipt of 0–2 antiretroviral agents compared to ART was associated with a relative risk of 2.9 (95% CI 1.3–6.7) for liver-related mortality [82]. More recently, analysis of 10,090 HIV/HCV coinfected individuals under observation in the United States Veterans Aging Study Virtual Cohort has again demonstrated benefits of ART at decreasing risk of disease progression [83]. Individuals initiating ART between 1996 and 2010 (defined as >3 agents from 2 or more classes) were assessed for incident hepatic decompensation. Overall the incidence rate for hepatic decompensation was 1.4/100 person-years. Individuals who initiated ART had a significantly reduced rate of hepatic decompensation relative to noninitiators (hazard ratio [HR] = 0.72; 95% CI 0.54–0.94). After accounting for potential confounding of undocumented ART at study entry the association became more pronounced (HR = 0.59; 95% CI, 0.43–0.82). Initiation of ART was found on average to be associated with a reduction in the rate of hepatic decompensation by 28%–41% [83]. Overall evidence derived from these and other cohort studies support ART-related decreases in fibrosis progression and potential reduction in liver-related mortality [84]. Nevertheless, a retrospective analysis of data from the Veterans Health Administration found that, despite ART-related virologic suppression, coinfected patients continued to have higher risk for hepatic decompensation compared to HCV monoinfected patients [85].

These data have been incorporated into current IAS-USA and US Department of Health and Human Services (DHHS), European and British treatment guidelines for HIV-infected individuals, where underlying hepatitis C coinfection is recognized as further justification to initiate ART irrespective of CD4 cell count [86–89].

In certain circumstances with CD4 cell counts >500 cells/*μ*L (i.e., in patients with significant baseline transaminitis) it may be reasonable, given the short duration of HCV therapy, to consider initiating HCV therapy first in order to decrease risk of ART-related hepatotoxicity and avoid drug-drug interactions between HCV antivirals and HIV antiretrovirals [90]. If therapy with the DAA regimen ombitasvir/paritaprevir/dasabuvir is being considered, ART initiation regardless of CD4 cell count is required as this DAA regimen contains ritonavir for pharmacotherapeutic boosting of paritaprevir and without the activity of a fully active ART regimen may promote HIV protease resistance-associated mutations in the untreated coinfected patients.

### 3.2. Antiretroviral Therapy and Risk of Hepatotoxicity in Coinfected Individuals

Hepatotoxicity is usually defined using the AIDS Clinical Trial Group (ACTG) grading system with grade 3 (ALT elevations greater than five times the upper limit of normal (ULN) range in individuals with normal values at baseline) considered a standard for more severe disease. Some experts have proposed an additional classification with grade 3 elevation considered as >3.5 × ULN when baseline values were abnormal [91]. Overall the incidence of hepatotoxicity in observational studies ranges from 2 to 18% [92], and the presence of HCV coinfection increases the risk by at least 2–5-fold [93–96]. Studies performed in the early ART era revealed increased risk of hepatotoxicity amongst coinfected individuals initiating ART containing the early protease inhibitors (PI), particularly high dose ritonavir [91, 93, 97], although other antiretrovirals with known hepatotoxicity profile such as the nonnucleoside reverses transcriptase inhibitors (NNRTI) nevirapine have also been implicated [98].

Tolerability of current first and second line NNRTI, PI, and integrase inhibitor agents in coinfected patients has been assessed in post hoc analysis of phases II and III randomized clinical trials including newer agents such as raltegravir [99], dolutegravir [100], rilpivirine [101], etravirine [102], and darunavir although relatively small numbers of coinfected individuals were included [103]. At present, there is limited information regarding use of the new boosted integrase inhibitor elvitegravir/cobicistat in coinfected patients, as HCV coinfection was identified in 5% of those randomized to this combination in trials when compared to efavirenz or atazanavir/ritonavir [104, 105]. Nonetheless, no significant hepatotoxicity was noted over 48 weeks of exposure.

Risk of antiretroviral-related hepatotoxicity has been associated with degree of underlying liver fibrosis. In a prospective study of 107 patients with biopsy-confirmed fibrosis ranging from F0 to F4, the overall incidence of hepatotoxicity was 5.1 events/100 person-years. However the incidence amongst those with F3 or F4 fibrosis was 38% compared to 15% in those with F1 or F2 fibrosis (RR 2.75; 95% CI 1.08–6.97) [106]. A potential association between ART and fibrosis progression has been observed in additional analyses [107, 108]. A direct causative association between ART and fibrosis progression in coinfected patients has not been well established and may be subject to additional confounders when assessed in terms of underlying alcohol or substance use, differing classes of antiretroviral agents, and the potential beneficial effects on hepatic disease progression associated with initiation of antiretroviral therapy as described above. Additionally, some studies suggest that genotype 3 infection may also be associated with increased risk for hepatotoxicity [109, 110]. Further evaluation of this potential interaction is required.

Successful HCV therapy has been associated with potential decrease in risk for subsequent antiretroviral-related hepatotoxicity [90]. In a cohort of 132 coinfected patients, sustained virologic response (SVR) following HCV therapy occurred in 33% of individuals. The yearly incidence rate of antiretroviral hepatotoxicity in those with SVR was 3.1% versus 12.9% in those without SVR [90].

At present, no specific antiretroviral regimen can be preferentially recommended for use in coinfected patients. However, certain regimens may need to be used cautiously in the setting of advanced liver disease. Close monitoring is required, and dosage adjustments or alterations of combination antiretroviral therapy may be required if hepatic decompensation occurs [87]. Certain antiretroviral agents must be avoided altogether due to drug-drug interactions when HCV therapy containing HCV protease inhibitors is being initiated (see Drug-Drug Interactions section below).

*Recommendations*

(4) ART regimens should be initiated as per current guidelines as they are effective and well-tolerated in coinfected patients (Class 1, Level A).
(5) Initiation of ART may serve to slow progression of liver disease in coinfected patients. Early initiation of ART is recommended for all individuals with CD4 >500 cells/*μ*L taking into account barriers to ART adherence and counselling regarding the long-term nature of ART (Class 1, Level B).
(6) All individuals being considered for therapy with the paritaprevir/ritonavir-based regimen should initiate ART prior to HCV therapy (Class 1, Level B).

## 4. Baseline Evaluation and Management of HCV in Coinfected Patients

Baseline evaluation and monitoring of coinfected patients is similar to that of monoinfected patients and should focus on determination of genotype and degree of liver disease/hepatic fibrosis as a prelude to consideration of HCV therapy (Table 2). Monitoring and treatment duration may be more intensive for patients with underlying cirrhosis, and steps to prevent HCV reinfection or infection with other viral hepatitis should be considered.

### 4.1. Diagnosis

In Canada, as many as 25%–30% of HIV-HCV coinfected persons are estimated to be unaware of their infection, highlighting the clear need for more HCV screening and testing [14]. Identification of HIV-HCV coinfection provides opportunities for preventing transmission, risk-reduction, counselling and linkage to care, and harm reduction services.

All HIV-infected patients should be screened for HCV coinfection by serologic testing. Similarly, all HCV-infected patients should be evaluated for HIV coinfection. In individuals with significant immune compromise, HCV antibodies may occasionally be falsely negative, and consideration should be given to directly testing for presence of HCV RNA [111]. False negatives are less common in the era of third generation HCV screening tests, but a high clinical index of suspicion should lead to direct nucleic acid testing for HCV RNA [112].

The frequency of HCV antibody testing should depend on ongoing risk behaviours (Section 1). Detection of HCV antibody does not determine active infection, as 10–25% of coinfected and monoinfected individuals will spontaneously clear the virus. The presence of HCV RNA should be confirmed after a positive HCV screening test to rule out spontaneous clearance by PCR. In EuroSIDA, 23% of anti-HCV positive individuals tested HCV RNA negative [62].

Individuals with positive HCV RNA should undergo determination of HCV genotype as an initial step of determining HCV therapy (Section 5).

Individuals with baseline negative HCV RNA should be considered for repeat testing to confirm the absence of chronic infection at least once, especially if ALT is elevated.

*Recommendations*

(7) Patients with confirmed HCV antibody should be evaluated with HCV RNA PCR (Class 1, Level C).
(8) Those with positive HCV RNA should undergo HCV genotyping (Class 1, Level C).
(9) Those with negative HCV RNA should undergo repeat testing at least once to confirm spontaneous clearance if liver enzymes are elevated (Class 1, Level C).

All individuals should also undergo screening for hepatitis A immunity (hepatitis A IgG) and for hepatitis B (HBsAg, anti-HBs, and anti-HBc) and should be vaccinated if nonimmune. If chronically infected with hepatitis B, they should be assessed for therapy.

*Recommendation*

(10) All patients should undergo screening for hepatitides A and B and should be offered vaccination if nonimmune (Class 1, Level C).

### 4.2. Clinical Assessment and Laboratory Monitoring

A detailed history and physical examination focused on signs and symptoms of liver disease is required. Features of advanced liver disease may include ascites, bulging flanks, peripheral edema, history of gastrointestinal bleeding, and jaundice. Examination includes assessment for splenomegaly, ascites, gynecomastia, spider nevi, and other manifestations of end-stage liver disease.

Monitoring of complete blood count (CBC), liver enzyme panel including ALT and AST, and markers of synthetic function (INR, albumin, and bilirubin) should be performed at baseline and can be monitored as a component of routine (every 6 months) laboratory testing in individuals undergoing ART therapy.

Thrombocytopenia may be a marker of hypersplenism and advanced liver disease. Derangements in synthetic function also suggest advanced disease. Caution should be used when interpreting elevated bilirubin levels in patients receiving atazanavir-based regimens as atazanavir is associated with unconjugated (indirect) hyperbilirubinemia, but elevated conjugated (direct) bilirubin levels indicate liver disease. Similarly, discordance between the absolute CD4 cell count and CD4 percentage (higher CD4 percentage than expected for the corresponding absolute value) in coinfected individuals may also suggest advanced disease. Amongst individuals enrolled in the CCC, 31% had evidence of high discordance, which was associated with markers of end-stage liver disease [113]. CD4 discordance has been shown also to correspond with advanced liver disease when assessed by transient elastography [114].

Additional baseline screen for other causes of chronic liver disease can be considered, including investigations for hemochromatosis (iron binding capacity with genetic testing if iron saturation exceeds 0.60), autoimmune hepatitis (including primary biliary cholangitis where appropriate, ANA, anti-smooth muscle antibody, anti-mitochondrial antibody, and immunoglobulin levels), Wilson's Disease (ceruloplasmin), and alpha-1-antitrypsin deficiency. Attention to alcohol consumption is essential given the negative influence alcohol has on fibrosis progression. Referral to alcohol cessation programs is a critical component to preserving liver health. Recognizing the diagnostic limitations, a workup for steatosis should be considered by performing a metabolic syndrome workup (lipid profile, glucose, and hemoglobin A1C), radiological evaluation (ultrasound, transient elastography-controlled attenuation parameter measurement), and potentially liver biopsy.

*Recommendations*

(11) Patients should be evaluated for other conditions which may result in or exacerbate chronic liver disease (Table 3) (Class 1, Level C).
(12) All patients should be counselled regarding alcohol reduction/abstinence and engaged in cessation programs when necessary (Class 1, Level C).

Ultrasound of the liver at baseline should also be considered and should be performed whenever there is thrombocytopenia. In cirrhotics, it should be conducted every 6 months for hepatocellular carcinoma screening [117].

Although liver enzyme elevations have traditionally been thought to reflect disease activity, it is now evident that HCV-infected individuals may develop fibrosis and even cirrhosis without significant liver enzyme elevations. In a retrospective review of 326 liver biopsies performed in coinfected individuals between 1997 and 2003 at a European centre, approximately 25% of individuals with persistently normal ALT values were found to have at least stage 2 fibrosis [118]. As such, ALT criteria alone should not determine treatment initiation in coinfected patients.

### 4.3. Role of Liver Biopsy

Liver biopsy has traditionally been regarded as the gold standard of investigation for HCV-related disease progression in North America [119]. The liver biopsy assesses both the degree of inflammatory activity and fibrosis and may also reveal an alternate etiology of liver damage. Nonetheless liver biopsies are invasive and difficult to repeat, often resulting in limited sample size and patient selection bias, so they are at best an imperfect gold standard. In addition, results may be affected by tissue sampling and interpretation error [120]. As noninvasive measures of liver fibrosis are now validated, liver biopsy should be reserved for instances where uncertainty about the stage of fibrosis remains after noninvasive assessment, in cases where multiple contributing factors to liver disease are being considered and/or where noninvasive technologies (i.e., transient elastography) are not available.

### 4.4. Noninvasive Assessment of Fibrosis: Transient Elastography and Laboratory Markers

Transient elastography (TE, Fibroscan*™*) is a noninvasive technique of measuring liver stiffness (with scores measured in kilopascal or kPa) which serves as a marker of hepatic fibrosis [121]. Use of TE for diagnosis of fibrosis has been established in a variety of chronic hepatic diseases including HCV [122]. Meta-analyses of TE compared to liver biopsy for assessment of fibrosis have found relatively high concordance, with one meta-analysis finding the mean areas under the receiver-operator curve (AUC) values for the diagnosis of significant fibrosis, severe fibrosis, and cirrhosis were 0.84, 0.89, and 0.94, respectively [123]. In another meta-analysis, the sensitivity and specificity of cutoffs for determining significant fibrosis were 71.9% and 82.4% and were 84.4% and 94.6% for cirrhosis [124].

TE has been validated in coinfected patients. In an analysis of 183 patients undergoing simultaneous liver biopsy, a cutoff of 7.2 kPa for significant fibrosis (>F2) had an area under the receiver-operator curve (ROC) of 0.83, while a cutoff of 12.5 kPa (ROC 0.95) was indicative of cirrhosis [115]. Similar ROC outcomes were obtained using these cutoffs in a cohort of coinfected patients [116]. Additional support for the use of TE was derived in a cohort of 169 Spanish patients undergoing liver biopsy, where the sensitivity and specificity of TE were evaluated [125]. To diagnose significant liver fibrosis, a cut-off value of 7.2 kPa was associated with a positive predictive value of 88% and a negative predictive value of 75%. To diagnose cirrhosis, a cut-off value of 14.6 kPa was associated with a positive predictive value of 86% and a negative predictive value of 94% [125]. Similarly, in an assessment of TE in a North American cohort of injection drug users (ALIVE Cohort), including coinfected patients, 79–83% of individuals, were correctly identified as having significant fibrosis and cirrhosis when compared to liver biopsy [126].

Fibroscan may be limited by body habitus (obesity may impair the ability of the probe to accurately assess the liver) and may be falsely elevated in circumstances of significant hepatic inflammation [121]. Of note, development of probes dedicated for use in obese patients may improve diagnostic value [127]. Recognizing that proposed values differ slightly from study to study, criteria for Fibroscan interpretation in coinfected patients are suggested in Table 3.

Use of noninvasive laboratory markers may aid in the assessment of fibrosis in coinfected patients. Use of the AST to platelet index (APRI) calculated as [(AST/ULN)/platelet count × 10^9^/L] × 100 has been validated in a Canadian cohort of coinfected patients [128, 129] where an APRI score of >1.5 was 100% specific and 52% sensitive for significant fibrosis compared to a gold standard of liver biopsy.

Other formulae for assessing fibrosis include the Fib-4 score (age [years] × AST [IU/L]/platelet count [expressed as platelets × 10^9^/L] × (ALT^1/2^ [IU/L]) [130] and FibroTest, a calculated algorithm of six serum tests (alpha-2-macroglobulin, apolipoprotein A1, haptoglobin, GGT, ALT, and bilirubin) with the age and sex of the patient [131, 132]. These methods lack sensitivity for diagnosing fibrosis when compared to TE [116, 133, 134].

### 4.5. Monitoring of Patients with Cirrhosis

Patients with confirmed cirrhosis should undergo additional monitoring for the development of complications such as hepatocellular carcinoma (HCC). Surveillance screening with regular ultrasounds (every 6 months) with or without use of serum alpha fetoprotein should be undertaken as is the case in HIV-negative individuals with cirrhosis. Referral to a gastroenterologist for consideration of endoscopy in order to screen and/or monitor esophageal varices may also be indicated.

Ongoing monitoring for HCC is also advised in patients with cirrhosis who have achieved SVR with HCV therapy, as the risk related to underlying cirrhosis may persist albeit diminished.

*Recommendations*

(13) ALT criteria alone should not be used to determine the need for treatment initiation in coinfected patients (Class 2a, Level C).
(14) Baseline abdominal ultrasound with Doppler should be considered in all patients (Class 2a, Level B).
(15) Baseline evaluation of liver fibrosis (e.g., Fibroscan, FibroTest, and APRI) to determine degree of hepatic fibrosis is advised (Class 2a, Level B).
(16) Evaluation of liver fibrosis with liver biopsy can be considered if noninvasive methods of determining fibrosis are not available, or if alternate diagnoses are being considered (Class 2a, Level C).
(17) Patients with evidence of underlying cirrhosis should be screened every 6 months for hepatocellular carcinoma using ultrasound (Class 1, Level B).
(18) Patients with underlying cirrhosis should be considered for gastroscopy for screening for esophageal varices (Class 1, Level B).

### 4.6. Preparation for HCV Therapy

Baseline laboratory determination of HCV status as outlined is necessary in order to evaluate HCV genotype and degree of hepatic fibrosis/disease.

Given the burden of comorbid conditions in the setting of coinfection, evaluation of factors such as substance use/addictions, mental health, and housing and food security is vital when preparing for HCV therapy. Substance use, lack of housing, or adequate food supply may limit the adherence to HCV therapy with deleterious effect on treatment outcome. However, if stabilized, these issues do not represent contraindications to treatment. In fact, HCV therapy can be successfully initiated and completed in active injection drug users [135]. Underlying mental health conditions may be exacerbated by interferon-based therapy, and use of noninterferon containing regimens should be strongly considered. Ongoing multidisciplinary follow-up is recommended.

Individuals considering HCV therapy should be assessed for potential contraindications. Contraindications include the following:

- Pregnancy (absolute contraindication based on either known teratogenicity of pegylated interferon/ribavirin or lack of data with DAA regimens).
- Decompensated liver disease (relative contraindication, particularly with pegylated interferon/ribavirin based regimens).

Individuals >50 years of age with history of hypertension, diabetes, or prior retinopathy should undergo baseline ophthalmology assessment if interferon-based therapy is considered, as it has been associated with exacerbation/new onset of retinopathy [136, 137].

When considering HCV therapy in PWID, concomitant use of harm reduction strategies is necessary given the risk of potential reinfection if injection drug use resumes after successful therapy [138]. Mathematical models suggest that HCV therapy in this population has the potential to reduce transmission within networks of IDU [139]. Individuals who have previously undergone successful HCV therapy should be reevaluated by HCV RNA testing if ALT elevation recurs to rule out reinfection.

#### 4.6.1. Adherence Management in the Era of DAA Therapy

Adherence is crucial to the success of treatment of HCV infection. In the era of interferon and ribavirin therapy, the consumption of 80% of each of the prescribed medications for a minimum of 80% of the target treatment duration was set as a “gold standard” [140], but such a standard has yet to be established in the era of DAA therapy. With respect to coinfection, a systematic review of factors influencing adherence to HCV therapy identified HIV coinfection as a positive influence [141], with an odds ratio of 2.52 (95% CI 1.36–4.67) in one study [142]. The authors suggest that this is related to better overall engagement in care.

Data on adherence in the DAA era are limited, but results from the NIAID SYNERGY trial in which “real-world” patients received combinations of 1–3 pills/day taken for 6–12 weeks suggest high levels of adherence in coinfected patients [143]. Adherence was monitored using MEMS caps. Overall, adherence exceeded 95% in almost all patients. Pill burden did have a modest effect (with those taking 3 versus 1 pills/day showing 94.8% versus 99.3% adherence) and adherence decreased from the first 4 (98.1%) to the last 4 (95.0%) weeks of treatment. Overall, 97% (58/60) of participants achieved SVR and there was no association between adherence and virologic nonresponse. It will be important with broader use of DAA-only therapy in clinical practice to ensure that such levels of adherence are maintained. In SYNERGY, the main reasons for nonadherence were working (39%), forgetting (35%), and being away from home (32%), factors which may be amenable to intervention.

Peer-driven support programs and multidisciplinary primary-care models have been used successfully for inner city populations of PWID [144, 145] and may serve to maximize the benefits of treatment by increasing adherence as well as long engagement in care to reduce the risk of reinfection in those at risk for this outcome. Once treatment is initiated, ongoing support and monitoring of adherence must be in place. The frequency of dispensing of HCV medications has been the subject of debate, at least partially related to the cost of therapy. Weekly dispensing of medications for populations with potential barriers to adherence (such as PWID) may be considered. Dispensing at longer intervals must be premised on demonstrated initial adherence and good tolerability of the treatment.

*Recommendations*

(19) All coinfected patients should undergo evaluation for HCV therapy (Class 1, Level A).
(20) Evaluation and management of factors such as substance use/addictions, mental health, and housing and food security are vital when preparing for HCV therapy (Class 1, Level B).
(21) Assuming appropriate supports are provided, addiction is not an exclusion criterion for HCV therapy (Class 1, Level B).
(22) Multidisciplinary care is recommended to optimally support patients as they progress through HCV workup and treatment (Class 1, Level B).
(23) If interferon will be used, detailed assessment for interferon-related contraindications is essential (Class 1, Level C).
(24) Appropriate levels of funding for HCV treatment programs and removal of barriers to HCV antiviral therapy are necessary to optimize engagement in care and treatment outcomes (Class 1, Level C). An adherence plan should be developed for all patients initiating HCV antiviral therapy (Class 1, Level C).

## 5. HCV Therapy in Coinfected Patients

There is clear evidence that successful HCV treatment leads to reduced disease burden from HCV infection. Successful treatment is the achievement of a sustained virological response (SVR), although historically this was defined as HCV RNA negativity at least 24 weeks after completion of antiviral therapy (SVR24); this is now defined as HCV RNA negativity at least 12 weeks after completion of antiviral therapy (SVR12) based on an FDA analysis [146]. SVR is equivalent to virological cure but does not confer immunity to reinfection. Successful HCV treatment has, to date, been the most effective means of preventing liver-related complications in the setting of HIV-HCV coinfection [147]. Despite this, a minority of persons has initiated treatment; only 1.1% (15 of 1360) initiated treatment for HCV from January 2000 to December 2004 in a BC inner city cohort of PWID [148]. In the CCC, 16% have been previously treated at the time of cohort enrolment baseline and 13% initiated treatment during follow-up (total: 29%). While being low, this is consistent with treatment rates reported in the literature elsewhere in the world, at least in the era prior to the availability of all oral therapy [149].

All coinfected patients should be assessed for HCV therapy. From 2001 until 2011, anti-HCV therapy consisted of pegylated interferon plus ribavirin (PR) for all HCV genotypes. The year 2011 heralded the availability of the first direct acting antiviral agents (DAAs) for HCV, boceprevir and telaprevir, both of which are HCV NS3 protease inhibitors. Boceprevir and telaprevir were approved only for genotype 1 in combination with PR. In late 2013, simeprevir, another NS3 protease inhibitor, was approved for use in combination with PR only in genotype 1. Then in December 2013, the uridine nucleotide NS5B polymerase inhibitor sofosbuvir was approved, leading to significant changes in recommended therapies, such that triple therapy with PR plus a NS3 protease inhibitor was no longer recommended as preferred therapy in genotype 1 and PR dual therapy was no longer recommended as preferred therapy in genotypes 2, 3, or 4. Additional new DAAs were approved in 2014 and 2015, and others are expected over the next two years.

At present, therapy for HCV is determined by HCV genotype. When treatment consisted of dual therapy with PR, SVR rates in the coinfected were significantly lower than in the HCV monoinfected, especially in genotype 1 [150–154], leading to the belief that HIV-HCV coinfected patients are harder to cure. However, in genotype 1, studies of triple therapy with PR plus an HCV NS3 protease inhibitor [155–157] or the nucleotide polymerase inhibitor sofosbuvir [158] demonstrated nearly identical SVR rates in the HIV-HCV coinfected compared with the HCV monoinfected. The combination of sofosbuvir plus ribavirin, while approved only for genotypes 2 and 3, has been studied in genotypes 1 to 4 in both the HCV monoinfected [159–161] and the HIV-HCV coinfected [158], and SVR rates are very similar.

### 5.1. Genotype 1 Treatment 

#### 5.1.1. Sofosbuvir-Ledipasvir

A fixed dose combination tablet containing sofosbuvir 400 mg and 90 mg of the NS5A inhibitor ledipasvir was approved in October 2014 for genotype 1, on the basis of three large clinical trials in the HCV monoinfected [162–164]. The clinical trials included 1952 patients with an overall SVR of 97%. The studies also showed that the addition of ribavirin was unnecessary and that ribavirin is associated with a higher incidence of anemia and poorer patient reported outcomes [165]. The duration of therapy is 12 weeks in most patients. However, 8 weeks is sufficient to achieve similarly high SVR rates in treatment-naïve noncirrhotic patients with baseline HCV RNA <6 million IU/mL [164]. Twenty-four weeks is necessary in treatment-experienced patients with cirrhosis [162]. Importantly, sofosbuvir-ledipasvir is as effective in persons who failed prior treatment with PR or PR plus a NS3 protease inhibitor as it is in the HCV treatment-naïve [162].

A 12-week regimen of sofosbuvir-ledipasvir was evaluated in 335 HIV-infected patients with HCV genotypes 1 and 4, of whom 20% had cirrhosis and 55% failed prior HCV therapy (ION-4) [166]. All patients were receiving ART with a TDF-FTC backbone accompanied by efavirenz, raltegravir, or rilpivirine. HIV protease inhibitors were not allowed. The SVR rate was 96% (321/335) in the whole study, 96% (313/327) with genotype 1, and 100% (8/8) with genotype 4. The SVR rate in genotype 1 is essentially the same as demonstrated in the HIV monoinfected population [162–164]. Amongst the 47 patients with cirrhosis who failed prior anti-HCV therapy, the SVR rate was 98% (46/47). This is higher than observed in HCV monoinfected treatment-experienced cirrhotics in which 24 weeks of sofosbuvir/ledipasvir and 12 weeks sofosbuvir/ledipasvir plus ribavirin achieved higher SVR rates than 12 weeks of sofosbuvir/ledipasvir [162, 163, 167, 168]. To date, there has been no evaluation of 8-week treatment duration in the HIV-HCV coinfected.

#### 5.1.2. Ombitasvir-Paritaprevir/Ritonavir Plus Dasabuvir and Ribavirin

The combination of the NS3 protease inhibitor paritaprevir boosted by the CYP3A4 inhibitor ritonavir, the NS5A inhibitor ombitasvir, and the NS5B nonnucleoside polymerase inhibitor dasabuvir, with ribavirin given for 12 weeks, results in SVR rates of 93 to 99% in HCV genotype-1 monoinfected patients, including PR treatment-experienced patients and those with compensated cirrhosis in multiple clinical trials [169–173]. The overall SVR of this regimen in the HCV genotype 1 monoinfected was 96% in 1376 patients in the phase 3 program. Ribavirin is needed in genotype 1a treatment but can be omitted in genotype 1b (in the absence of cirrhosis) [171]. Paritaprevir, ombitasvir, and ritonavir are coformulated and are given as two coformulated tablets once daily. Dasabuvir is dosed separately twice daily. The pill burden of this regimen is 6 per day with ribavirin and 4 per day without ribavirin.

This regimen (including ribavirin) was evaluated in 63 patients coinfected with HIV and HCV genotype 1 [174]. Two-thirds were HCV treatment-naïve, one-third failed prior PR therapy, and 19% had cirrhosis. Thirty-one study participants were treated for 12 weeks and 32 were treated for 24 weeks. SVR rates were 94% (19/31) with 12 weeks and 91% (29/32) with 24 weeks of therapy. Participants could only receive raltegravir (*n* = 35) or atazanavir (*n* = 28) as “HIV anchor drugs” in combination with two HIV nucleoside reverse transcriptase inhibitors.

The presence of multiple CYP3A4 metabolized medications, including ritonavir, limits antiretroviral treatment options in HIV coinfected patients considered for this regimen. Specifically, it is not recommended to administer efavirenz, rilpivirine, etravirine, or lopinavir/ritonavir, darunavir/cobicistat with this regimen. Darunavir Cmin is reduced by approximately 50% with this regimen. The clinical significance of this reduction in darunavir exposure is unknown, but caution should be exercised. This regimen is not recommended for patients who failed PR plus a NS3 protease inhibitor because of the concern that NS3 protease resistance mutations will compromise the activity of paritaprevir and the absence of clinical data in this patient population.

Due to concern regarding hepatotoxicity this regimen in contraindicated in those with decompensated liver disease [Holkira PM] [175].

#### 5.1.3. Sofosbuvir-Simeprevir

In the COSMOS study, 167 HCV genotype 1 monoinfected, treatment-naïve, and prior PR null responders (i.e., failure to achieve a 2 log reduction in HCV RNA by week 12 of PR treatment) received once daily sofosbuvir plus simeprevir (a NS3 protease inhibitor), with (*n* = 108) or without (*n* = 59) ribavirin for either 12 (*n* = 82) or 24 weeks (*n* = 85) [176]. In the first cohort of 80 null responders to prior PR with METAVIR F0–F2 disease, SVR12 rates with dual therapy were high at 92-93% after 12 or 24 weeks of therapy, and the addition of ribavirin was not clearly associated with improvement in SVR rates although the study was not powered to demonstrate statistical noninferiority [176]. For the second cohort of 87 naïve and null responders with METAVIR F3-F4 fibrosis, SVR12 rates were 93% with 12 weeks of therapy and 96% with 24 weeks of therapy. The addition of ribavirin did not increase SVR rates but did result in some cases of anemia. On the basis of the COSMOS data, two phase 3 studies, evaluated 8 versus 12 weeks of sofosbuvir plus simeprevir in noncirrhotics (OPTIMIST-1) [177] and 12 weeks in cirrhotics (OPTIMIST-2) [178] in HCV genotype 1 monoinfected treatment-naïve participants. OPTIMIST-1 confirmed high SVR rates with 12 weeks of therapy (97% in the treatment-naïve and 95% in the treatment-experienced), but SVR rates were suboptimal with 8 weeks of therapy (85% in the treatment-naïve and 77% in the treatment-experienced). The efficacy of 12 weeks of sofosbuvir plus simeprevir in participants with compensated cirrhosis was 88% in the treatment-naïve and 79% in the treatment-experienced [OPTIMIST-2]. At present, minimal data exist for this combination in coinfected individuals.

#### 5.1.4. Sofosbuvir-Daclatasvir

Daclatasvir was the first NS5A inhibitor to be studied in combination with sofosbuvir. In a phase 2 study in the HCV monoinfected, SVR rates after 24 weeks of treatment with sofosbuvir plus daclatasvir with or without ribavirin were 98% in genotype 1 (*n* = 85), 92% in genotype 2 (*n* = 26), and 89% in genotype 3 (*n* = 18) [179]. An additional arm of 12 weeks was added in genotype 1 and the SVR rate was 94% (*n* = 82). While not statistically powered to assess the contribution of ribavirin, it did not appear that ribavirin increased SVR rates.

A phase 3 study of sofosbuvir plus daclatasvir without ribavirin was recently completed in 203 HIV coinfected participants of which 168 were genotype 1 infected [180]. HCV treatment-naïve participants were randomized 2 : 1 to 12 versus 8 weeks of therapy and treatment-experienced participants were given 12 weeks 12 of therapy. SVR rates in genotype 1 infection were excellent with 12 weeks of therapy, being 96% in treatment-naïve (*n* = 83) and 98% in treatment-experienced participants (*n* = 44), but were disappointing with 8 weeks of treatment, at 76%. The 12-week arm yielded SVR rates of 96% in genotype 1a (*n* = 104) and 100% in genotype 1b (*n* = 23). SVR rates in the overall study population were slightly lower in cirrhotic patients (91.7%, *n* = 24) than in noncirrhotic patients (98.4%, *n* = 124).

#### 5.1.5. Grazoprevir-Elbasvir

Grazoprevir is a protease inhibitor and elbasvir is an NS5a inhibitor. This once daily regimen combination received regulatory approval in early 2016. In the C-EDGE COINFECTION study, 218 HIV-HCV coinfected study participants with genotype 1, 4, or 6 infection naïve to HCV treatment received this regimen for 12 weeks [181]. Safety and tolerability were excellent and the overall SVR rate was 96% an additional first line regimen for genotype 1 management.

*Recommendations*

(25) * Genotype 1 Treatment-Naïve Individuals*
  First Line: sofosbuvir 400 mg coformulated with ledipasvir 90 mg daily for 12 weeks of therapy (Class 1, Level B), or
   ombitasvir/paritaprevir/ritonavir plus dasabuvir plus ribavirin for 12 weeks or grazaprevir-elbasvir for 12 weeks (Class 1, Level C).
(26) * Genotype 1 Treatment-Experienced Patients*
  First Line: sofosbuvir 400 mg daily coformulated with ledipasvir 90 mg daily for 12 weeks (Class 1, Level B). Cirrhotic patients should be treated for 24 weeks, based on data in the HCV monoinfected (Class 1, Level C), or
   ombitasvir/paritaprevir/ritonavir plus dasabuvir plus ribavirin for 12 weeks (Class 1, Level B) (this regimen may be used in patients who failed dual PR therapy but is not recommended for patients who have failed a regimen including a NS3 protease inhibitor).
(27) * Regimens No Longer Recommended for First Line Use*
  1. Triple therapy with PR plus any DAA for HCV genotype 1 is no longer recommended for use given the improved efficacy, safety, and tolerability profiles of all oral therapy.
  2. Sofosbuvir plus simeprevir for genotype 1 for reasons of cost and minimal data in the coinfected.

### 5.2. Genotypes 2 and 3: Data in HCV Monoinfection

Sofosbuvir plus ribavirin has been evaluated for use in genotypes 2 and 3 in a large noninferiority trial with standard pegylated interferon/ribavirin as the comparator [182]. In the FISSION trial, 499 treatment-naïve individuals were randomized to 12 weeks of therapy with sofosbuvir/ribavirin or 24 weeks of pegylated interferon/ribavirin. Individuals with genotype 2 infection had exceptional SVR rates of 97% with sofosbuvir/ribavirin versus 76% with pegylated interferon/ribavirin, while those with genotype 3 achieved similar SVR rates to pegylated interferon/ribavirin (56% versus 63%). Cirrhosis markedly reduced SVR rates for genotype 3 individuals to approximately 30% in both arms. Similar SVR rates were seen in the POSITRON trial in interferon-ineligible participants [183]. In the phase III VALENCE study, improved SVR rates were seen in genotype 3 treatment-naïve individuals who received 24 weeks of sofosbuvir/ribavirin with SVR rates of 94%, with the subgroup of cirrhotic patients achieving SVR rates of 90% [160].

Sofosbuvir plus ribavirin has also been evaluated in treatment-experienced genotypes 2 and 3 participants. In the FUSION trial, individuals were randomized to receive 12 or 16 weeks of therapy with sofosbuvir and ribavirin. Those with genotype 2 achieved an SVR rate of 86% after 12 weeks and 94% after 16 weeks. SVR rates were much lower for genotype 3, with an SVR rate of 30% in those receiving 12 weeks versus 62% in those who received 16 weeks of therapy [183]. In the VALENCE study, treatment-experienced genotype 2 patients experienced similar high rates of response (91%) after 12 weeks of therapy of dual therapy. Treatment-experienced patients with genotype 3 treated with 24 weeks of sofosbuvir and ribavirin achieved an SVR of 87% in those without cirrhosis and only 60% in those with cirrhosis [160].

In the LONESTAR-2 phase II trial, the addition of pegylated interferon to a 12-week course of sofosbuvir/ribavirin resulted in SVR rates of 83% for genotype 3, with or without cirrhosis [184]. The BOSON study evaluated a 12-week regimen of pegylated interferon plus ribavirin plus sofosbuvir compared to 16 or 24 week durations of sofosbuvir plus ribavirin [185]. Five hundred and forty-four study participants with genotype 3 monoinfection and 48 treatment-experienced cirrhotic patients with genotype 2 monoinfection were evaluated in this study. In genotype 3, SVR rates were numerically highest with the 12-week triple regimen (93%) compared with the 24-week regimen of sofosbuvir plus ribavirin (84%) and were also higher in all subgroups (cirrhotics, noncirrhotics, treatment-naïve, and treatment-experienced).

A 12-week regimen of sofosbuvir plus daclatasvir was highly efficacious in HCV genotype 3 monoinfected patients without cirrhosis, whether HCV treatment-naïve (SVR 97%) or experienced (SVR 94%), but was much less effective in the presence of cirrhosis (SVR 58% in the treatment-naïve and 69% in the treatment-experienced) [186]. Of 36 compensated cirrhotics, SVR rates were 83% with 12 weeks and 89% with 16 weeks. In treatment-experienced cirrhotics, SVR rates were 88% with 12 weeks and 86% with 16 weeks. All 14 participants with stage 3 fibrosis achieved SVR.

#### 5.2.1. Data in HIV-HCV Coinfected Patients

Sofosbuvir was evaluated in HIV coinfected patients in the phase 2 Study P7977-1910 trial [158]. In this open-label study, 23 coinfected treatment-naïve study participants received sofosbuvir 400 mg daily in conjunction with pegylated interferon and weight-based ribavirin for 12 weeks. Individuals were predominantly genotype 1 infected, with two individuals with genotype 3, and a single individual with genotype 2 and 4, respectively, were also enrolled. The ART regimens included efavirenz, rilpivirine, raltegravir, and the boosted protease inhibitors atazanavir and darunavir. Overall, the SVR12 was 91%. Side effects were predominantly those of pegylated interferon and ribavirin.

In the phase III PHOTON-1 study, three cohorts of coinfected patients (genotype 1 treatment-naïve patients *n* = 114, genotypes 2 (*n* = 28) and 3 (*n* = 42) naïve patients, and genotypes 2/3 treatment-experienced patients (*n* = 41) were enrolled to receive either 12 weeks or 24 weeks (genotype 1 and treatment-experienced patients) of sofosbuvir with ribavirin [187]. Individuals could be on a wide range of ART regimens due to the lack of drug interactions, or naïve to ART if baseline CD4 cell count was >500 cells/mm^3^. The majority of those enrolled were on ART, receiving predominantly efavirenz, atazanavir, or darunavir-based regimens. The SVR24 rate was 75% for genotype 1 participants, 88% for genotype 2, and 67% for genotype 3 patients. Amongst treatment-experienced patients, SVR24 was attained by 92% of genotype 2 and 88% of genotype 3 individuals. Overall, the regimen was well tolerated, with more adverse events related to sofosbuvir/ribavirin seen in those receiving a 24-week course of therapy.

PHOTON-2 was a multicentre phase 3 study of sofosbuvir and ribavirin in HIV/HCV coinfected patients [188]. Nineteen treatment-naïve and 6 treatment-experienced genotype 2, 57 treatment-naïve and 49 treatment-experienced genotype 3, and 31 treatment-naïve genotype 4 patients participants were enrolled in the study and completed treatment. Thirty-seven (23%) of the nongenotype 1 patients were cirrhotic. Almost all participants were on ART with CD4 T cell counts in all groups greater than 200 and medians between 499 and 633. SVR12 was 89% in treatment-naïve genotype 2, and 83% in treatment-experienced genotype 2. SVR12 in genotype 3 was similar, 91% in treatment-naïve, and 86% in treatment-experienced individuals. Adverse events were rare, and the discontinuations due to adverse events were 2-3% in all nongenotype 1 groups.

Ledipasvir has little genotype 2 activity and is not recommended for use in coinfected patients. Data from 51 treatment-naïve, mostly noncirrhotic patients in the phase 2 ELECTRON-II study, suggests there may be a role for sofosbuvir, ledipasvir, and ribavirin for 12 weeks in HCV genotype 3 treatment-naïve, noncirrhotic, and HCV monoinfected individuals [189]. However, these data are too preliminary to recommend the combination over other regimens.

Overall, while the number of treated patients is low in the dedicated coinfected studies, these data do suggest that genotypes 2 and 3 can be treated with high cure rates with sofosbuvir and ribavirin, in some circumstances combined with ledipasvir (HCV GT3) or pegylated interferon. However, it is worth noting that other potent, all oral combinations may soon be available for HCV genotypes 2 and 3 treatment and that delay of treatment in those who do not have an urgent need for treatment is reasonable.

A phase 3 study of sofosbuvir plus daclatasvir (without ribavirin) in the HIV coinfected included mainly genotype 1 infected participants (83%) but also included a small number with nongenotype 1 infection (ALLY-2 trial) [180]. The 12-week treatment duration arm yielded SVR rates of 100% (G2, *n* = 13; G3, *n* = 10; G4, *n* = 3). There were minimal numbers of HIV-HCV coinfected genotypes 2 and 3 infected cirrhotics (treatment-naïve and treatment-experienced) to draw definitive conclusions regarding the efficacy of 12-week duration, ribavirin-free treatment in these subpopulations.

*Recommendations*

(28) * Genotype 2 Treatment-Naïve Patient*
  First line: sofosbuvir 400 mg daily with weight-based ribavirin for 12 weeks with consideration for 16 weeks in cirrhotics (Class 1, Level B).
  Second line: sofosbuvir 400 mg daily and Daclatasvir 60 mg daily for 12 weeks (Class 1, Level B).
(29) * Genotype 2 Treatment-Experienced Patient*
  First line: sofosbuvir 400 mg daily with weight-based ribavirin for 12 weeks, or 16 weeks in cirrhotics (Class 1, Level B).
  Second line: sofosbuvir 400 mg daily with weight-based ribavirin and pegylated interferon 180 *μ*g weekly for 12 weeks (Class 1, Level B), or
   sofosbuvir 400 mg daily and Daclatasvir 60 mg daily for 12 weeks (Class 1, Level B).

*Recommendations for Treatment-Experienced Coinfections Are Based on Expert Recommendation*,* Utilizing Limited Data in Coinfection*,* and Extrapolation from Data in Monoinfected Populations*

(30) * Genotype 3 Treatment-Naïve Patient*
  First line: * * sofosbuvir 400 mg daily with weight-based ribavirin and pegylated interferon 180 *μ*g weekly for 12 weeks (Class 1, Level B), or
   sofosbuvir 400 mg daily with weight-based ribavirin for 24 weeks (noncirrhotics) (Class 1, Level B), or
   sofosbuvir 400 mg daily and Daclatasvir 60 mg daily for 12 weeks (noncirrhotics) and sofosbuvir 400 mg daily and Daclatasvir 60 mg daily with weight-based ribavirin for 12 weeks (Cirrhotics) (Class 1, Level B).
(31) * Genotype 3 Treatment-Experienced Patient*
  First line: sofosbuvir 400 mg daily with pegylated interferon alpha 2a 180 *μ*g weekly and ribavirin for 12 weeks (Class 1, Level C), or
   sofosbuvir 400 mg daily with weight-based ribavirin for 24 weeks (Class 1, Level B), or
   sofosbuvir 400 mg daily and Daclatasvir 60 mg daily for 12 weeks (noncirrhotics) and sofosbuvir 400 mg daily and Daclatasvir 60 mg daily with weight-based ribavirin for 12 weeks (cirrhotics) (Class 1, Level B).

#### 5.2.2. Genotypes 4–6

There are limited data on direct acting antiviral therapy in HCV genotypes 4, 5, and 6 infected patients as few studies, dedicated or otherwise, have included them, and when permitted were conducted in countries with a low prevalence of genotypes 4–6 infection. Data in these genotypes are even rarer in the context of HIV/HCV coinfected individuals.

Eight HIV-HCV coinfected, genotype 4 patients received sofosbuvir 400 mg daily coformulated with ledipasvir 90 mg daily for 12 weeks in the ION-4 study [166]. All achieved an SVR.

The all oral, 12-week regimen ombitasvir, paritaprevir, ritonavir, with or without ribavirin was assessed in 135 treatment-naïve and experienced, noncirrhotic HCV genotype 4 monoinfected patients in the PEARL-I study [190]. In the 86 treatment-naïve patients, SVR12 was 100% in the ribavirin containing arm and 91% without ribavirin, although the difference was not statistically significant. All 49 treatment-experienced individuals achieved SVR12.

The NEUTRINO study treated HCV monoinfected patients with sofosbuvir, pegylated interferon, and ribavirin for 12 weeks and included a small number of genotype 4 (30 patients) and genotype 5 or 6 (7 patients). 96% of genotype 4 patients and all of the 7 genotype 5 or 6 patients achieved SVR12 in this study [182].

The PHOTON-2 trial also included 31 HCV genotype 4, treatment-naïve patients treated for 24 weeks with sofosbuvir and weight-based ribavirin with 84% of patients achieving SVR12 [188]. Two other small studies assessed sofosbuvir and ribavirin in the treatment of HCV genotype 4 monoinfection. A phase 2 study of sofosbuvir and ribavirin for 12 or 24 weeks in 60 treatment-naïve and treatment-experienced Egyptian HCV genotype 4 patients demonstrated SVR12 in 68% of the 12 week group and 93% of the 24 week group [161].

The SYNERGY trial treated 21 individuals with genotype 4 with 12 weeks of sofosbuvir and ledipasvir, 33% of whom had compensated cirrhosis, with an SVR12 in 95% of those treated [143, 191].

(32) * Genotype 4 Treatment-Naïve and Experienced*
  First line: sofosbuvir 400 mg daily and ledipasvir 90 mg daily for 12 weeks (Class 1, Level B), or
   paritaprevir 150 mg daily, ritonavir 100 mg daily, ombitasvir 25 mg daily, and weight-based ribavirin for 12 weeks (Class 1, Level C), or
   sofosbuvir 400 mg daily with pegylated interferon and ribavirin for 12 weeks (NB-based on HCV monoinfection studies) (Class 1, Level C), or
   sofosbuvir 400 mg daily and weight-based ribavirin daily for 24 weeks.

There are currently insufficient data in HIV-HCV coinfection with genotypes 4–6 to comment on the efficacy of sofosbuvir-simeprevir or sofosbuvir-daclatasvir. Likewise, there are currently insufficient data in HIV-HCV coinfection with genotypes 5-6 to comment on the efficacy of sofosbuvir with pegylated interferon and ribavirin.

## 6. Salvage Therapy for DAA Treatment Failures

### 6.1. Failure of NS3 Protease Inhibitor Plus Peginterferon and Ribavirin

Studies of triple therapy with peginterferon plus ribavirin plus a NS3 protease inhibitor demonstrate that over 80% of failures are associated with treatment-emergent resistance mutations in the NS3 region [192–195]. Over time, these resistance mutations revert back to wild-type, with reversion occurring more quickly in subgenotype 1b than in subgenotype 1a [196].

As noted earlier, the ION-2 study of sofosbuvir/ledipasvir with or without ribavirin in 440 patients with genotype 1 monoinfection who failed prior therapy included 231 patients (52.5% of the study population) who failed prior therapy with peginterferon plus ribavirin plus a NS3 protease inhibitor and 209 who failed prior dual therapy with peginterferon plus ribavirin. SVR rates were over 93% and virtually identical in both groups of patients, and the addition of ribavirin did not improve SVR rates [162].

As noted earlier, a 12-week regimen of sofosbuvir/ledipasvir was evaluated in 335 HIV coinfected patients of whom 98% had HCV genotype 1 infection and 2% had HCV genotype 4 infection [166]. Fifty-three patients in ION-4 failed a previous regimen of peginterferon plus ribavirin plus a NS3 PI, of whom 52 (98%) achieved SVR.

The regimen of paritaprevir/ombitasvir/ritonavir plus dasabuvir with or without ribavirin has not been evaluated in patients who failed therapy including a NS3 protease inhibitor. Indeed, studies of this regimen that included prior treatment failures specifically excluded patients who had received NS3 protease inhibitors because of concerns that NS3 resistance mutations might compromise the efficacy of paritaprevir [172, 173].

In an open-label “real life” cohort study of patients treated with sofosbuvir plus simeprevir, patients who failed prior therapy with boceprevir or telaprevir had a lower SVR rate (76%; 35/46) than those with no prior NS3 protease inhibitor exposure (SVR rate 93%; 118/129), suggesting that prior NS3 protease inhibitor exposure compromises the activity of simeprevir [197].

### 6.2. Failure of Sofosbuvir Plus Ribavirin

Twenty patients with HCV genotype 1 monoinfection who failed treatment with sofosbuvir plus ribavirin were treated with a 12-week course of sofosbuvir/ledipasvir plus ribavirin and all achieved SVR [198]. Thirteen HIV-HCV coinfected patients who relapsed after treatment with sofosbuvir plus ribavirin in the PHOTON-1 study all achieved SVR after 12 weeks of sofosbuvir/ledipasvir therapy in ION-4 [166].

### 6.3. Failure of Peginterferon Plus Ribavirin Plus Sofosbuvir

Twenty-five patients with HCV genotype 1 monoinfection who failed treatment with peginterferon plus ribavirin plus sofosbuvir were treated with a 12-week course of sofosbuvir/ledipasvir plus ribavirin and all achieved SVR [198].

### 6.4. Failure of Sofosbuvir/Ledipasvir

Virological failure is very uncommon in HCV genotype 1 infection treated with sofosbuvir/ledipasvir. In a combined analysis of 2144 patients from the ION 1–3, LONESTAR and ELECTRON studies, virological failure occurred in only 2.4% (51/2144) of patients [199]. At virological failure, 39 patients (76%) had resistance-associated variants (RAVs) in NS5A and only 3 had RAVs in NS5B, of which only one had S282T. Unlike NS3 RAVs, which revert over time, NS5A RAVs persist for at least two years [200].

Limited data are available regarding retreatment of patients who failed sofosbuvir/ledipasvir therapy. The single patient noted above whose virus developed the S282T NS5B RAV was retreated with sofosbuvir/ledipasvir plus ribavirin for 24 weeks and achieved SVR.

Forty-one patients with HCV genotype 1 infection who failed 8- or 12-week courses of sofosbuvir/ledipasvir were retreated with a 24-week course of sofosbuvir and ledipasvir and SVR was achieved in 71% (29/41) [201]. SVR was 100% in the 11 patients without NS5A RAVs, but only 60% in the 30 patients with NS5A RAVs. Only 2 of 6 patients with Y93H/N NS5A RAVs, which are associated with high-level in vitro resistance, achieved SVR.

When patients fail treatment with sofosbuvir/ledipasvir, most will have NS5A RAVs and almost none will have NS5B RAVs [162–164, 202, 203]. Therefore, retreating with sofosbuvir in combination with a DAA that does not target NS5A is a strategy that makes sense, although it has not been studied in this setting. However, in HCV treatment-naïve patients, the presence of NS5A RAVs had no effect on SVR rates with sofosbuvir plus the protease inhibitor simeprevir, supporting this concept [177, 178].

### 6.5. Failures of Sofosbuvir Plus Simeprevir

There are no data yet available on retreatment of patients failing sofosbuvir plus simeprevir. Nevertheless, data indicate that patients failing this regimen generally have viruses with treatment-emergent NS3A RAVs but no NS5B RAVs [176], so it is likely that sofosbuvir/ledipasvir would be effective [177, 178].

### 6.6. Failures of Paritaprevir/Ombitasvir/Ritonavir Plus Dasabuvir with or without Ribavirin

There are no data yet available on retreatment of patients failing paritaprevir/ombitasvir/ritonavir plus dasabuvir with or without ribavirin, which occurs very infrequently. Pooled phases 2 and 3 data of 1083 patients treated with the recommended regimen identified 19 cases of virological failure (1.8%), of which 18 were of genotype 1a. At virological failure, 83% had NS3A RAVs, 78% had NS5A RAVs, and 78% had NS5B RAVs [204]. It is expected that sofosbuvir will remain active against such strains of HCV, but there is no predictably active “partner” DAA. If patients failing paritaprevir/ombitasvir/ritonavir plus dasabuvir with or without ribavirin must be treated in the absence of data regarding a known effective regimen, it is suggested that triple therapy with sofosbuvir plus peginterferon plus ribavirin for 12 weeks be considered, provided that interferon is not contraindicated.

## 7. Drug-Drug Interactions

### 7.1. Direct Acting Antivirals and Antiretrovirals

The potential for interactions between HCV directly acting antiviral agents and other drug classes is high due to the pharmacological characteristics of these HCV agents, particularly in the context of earlier ART initiation, the aging HIV population, and need for management of comorbidities [205–207].

HIV protease inhibitors, nonnucleoside reverse transcriptase inhibitors, and the integrase inhibitor elvitegravir are substrates and inhibitors or inducers of numerous cytochrome P 450 (CYP450) hepatic enzymes and transporters. The integrase inhibitor raltegravir is not a P450 substrate, whereas for dolutegravir it represents only a minor pathway. Neither raltegravir nor dolutegravir are inducers or inhibitors of these enzymes and therefore may be used with HCV DAAs without dosage adjustment [208, 209]. Underlying HIV resistance mutations may compromise HIV suppression if individuals are switched from a robust protease inhibitor-based regimen to raltegravir to accommodate DAA use. Regimen switches of this nature must take into account prior HIV therapies [210]. Dolutegravir appears to have a higher genetic barrier to resistance than raltegravir [211]. Of note, the integrase inhibitor elvitegravir is a CYP3A4 substrate and is coformulated with the pharmacokinetic booster cobicistat, making it more prone to interactions than the other integrase inhibitors. The CCR5 inhibitor maraviroc and the NNRTI rilpivirine are CYP3A4 substrates but do not exert inhibiting or inducing effects on the P450 system.

Similarly, the newer HCV agents are also substrates and inhibitors or inducers of various P450 enzymes and transporters. The NS5A inhibitor ledipasvir (which is coformulated with sofosbuvir) is a substrate and weak inhibitor of P-glycoprotein (P-gp). It is also a weak inhibitor of transporters including BCRP (breast cancer resistance protein) and OATP (organic anion transporter protein) 1B1/1B3.

Ledipasvir/sofosbuvir may be coadministered with most antiretrovirals, but special attention is required for tenofovir-containing regimens. Tenofovir exposures are increased 40–98% in the presence of ledipasvir/sofosbuvir, regardless of the type of antiretroviral combination used. This effect is postulated to be secondary to inhibition of P-gp and BCRP-mediated efflux of tenofovir by ledipasvir. There is no evidence to suggest that clinical relevant negative consequences result from this interaction. Nonetheless, patients continuing on tenofovir treatment during ledipasvir/sofosbuvir therapy should be monitored for tenofovir-associated adverse events. Use of an alternate NRTI backbone may be considered, particularly in patients with additional risk factors for renal dysfunction including use of other potentially nephrotoxic agents (including NSAID use), or when prolonged ledipasvir/sofosbuvir treatment (i.e., greater than 12 weeks) is required.

The combination of paritaprevir and ombitasvir, both of which are substrates of CYP3A4, P-glycoprotein, and BCRP, is combined also with dasabuvir, a substrate of CYP2C8, 3A4, P-glycoprotein, and BCRP. Paritaprevir and ombitasvir are coformulated with ritonavir since paritaprevir requires pharmacokinetic boosting for optimal exposures. This coformulated regimen should not be given with NNRTIs or certain boosted antiretrovirals due to risk of altered DAA exposures and/or increased risk of adverse events.

The NS5A inhibitor daclatasvir is a substrate of CYP3A4 and P-gp and is an inhibitor of P-gp, OATP1B1, OCT1, and BCRP. Daclatasvir requires dose adjustment with certain boosters and enzyme inducers.

The investigational combination of grazoprevir and elbasvir has recently been filed for evaluation by the FDA and Health Canada and is anticipated to be available as a fixed dose, once daily combination product in early 2016. Grazoprevir is an NS3/4A protease inhibitor and a substrate of CYP3A4, P-gp, and OATP1B1. Elbasvir is an NS5A inhibitor and is a substrate of CYP3A4, P-gp, and OATP. Grazoprevir inhibits CYP2C8 and is a weak inhibitor of 3A4 and UGT (uridine glucuronosyltransferase) 1A1. Both grazoprevir and elbasvir inhibit the BCRP transporter. From a practical standpoint, grazoprevir/elbasvir are mainly susceptible as victims of drug-drug interactions (DDIs) rather than perpetrators. Grazoprevir/elbasvir should not be coadministered with boosted protease inhibitors or efavirenz due to significant increases or decreases in DAA concentrations [212–215]. Grazoprevir may be safely coadministered with raltegravir, dolutegravir, or rilpivirine [216–218]. Of note, due to effects on BCRP, rosuvastatin (but not pravastatin) concentrations are significantly increased with grazoprevir/elbasvir, and caution is recommended with this combination [219].

Therefore, there is a high potential for drug interactions in the coinfected population, particularly if simultaneous treatment of HCV and HIV is required.

Negative consequences of drug interactions include HIV and HCV viral breakthrough and development of resistance, suboptimal disease/symptom management, or drug toxicities and possible nonadherence [220]. A recent study assessed the risk of potential drug interactions in an HIV-HCV coinfected population with first line HCV regimens including simeprevir, ledipasvir/sofosbuvir, and paritaprevir/ritonavir. In up to 76% of patients, a change in antiretroviral therapy would be required in order to accommodate initiation of HCV treatment. However, due to underlying HIV resistance, antiretroviral regimen changes were not feasible in a significant proportion of patients [221]. These results highlight the need for involving clinicians and pharmacists experienced in both HIV and HCV in order to ensure optimal management of both conditions. A summary of potential and demonstrated pharmacokinetic interactions between ARVs and DAAs is included in Tables 4 and 5.

*Recommendations*

(33) Careful attention to drug-drug interactions between HCV antivirals and concurrently administered HIV and non-HIV medications is critical to avoid viral breakthrough of either HIV or HCV, development of resistance, suboptimal disease/symptom management, and drug toxicities (Class 1, Level C).
(34) For individuals with genotype 1 infection initiating therapy with sofosbuvir/ledipasvir, traditional first line antiretrovirals may be used. If a tenofovir-based regimen is used, close monitoring of renal function is recommended due to potential for increased tenofovir exposures (Class 2b, Level B).
(35) For individuals with genotype 1 infection initiating HCV therapy with ombitasvir/paritaprevir/ritonavir plus dasabuvir, atazanavir (without additional booster), raltegravir, or dolutegravir may be used (Class 2b, Level B).
(36) For individuals with genotype 1 infection initiating HCV therapy, switch from alternate regimens to an acceptable regimen as listed above can be considered if HIV treatment history and resistance profile permits such a switch.
(37) For patients with HIV multidrug resistance who are well controlled on nonpreferred ART regimens, initiation of triple therapy including DAAs may be considered in consultation with an expert physician and pharmacist with experience in managing HIV and HCV drug interactions.

### 7.2. Interactions between DAAs and Other Drug Classes

Many common drugs from multiple different classes are at risk of drug interactions with DAAs. The product monographs of direct acting agents provide a list of drugs with known or potential CYP interactions. In addition to CYP450 isoenzymes, drug transporters including OATP1B1/3, P-gp, and BCRP are responsible for changes in drug disposition. Drugs altering gastric pH may also lead to clinically relevant drug interactions. For instance, ledipasvir requires an acidic environment for optimal absorption. Examples of interacting drug classes include acid-reducing agents, benzodiazepines (e.g., midazolam), HMG coenzyme A reductase inhibitors (statins), macrolides, rifamycins (e.g., rifampin), anticonvulsants, antiarrhythmics, psychotropics, azole antifungals, erectile dysfunction drugs, antipsychotics, inhaled corticosteroids, calcium channel blockers, immunosuppressants, and more. Herbals and over-the-counter drugs are not exempt of potential significant interactions. St-John's wort (*Hypericum perforatum*) is the most cited interacting drugs due to induction of CYP450 and drug transporters. Other herbals such as Gingko Biloba, garlic, and American ginseng were also shown to increase the clearance of other drugs [222]. Interactions related to drug transporters are not as well characterized as traditional interactions through CYP450 or glucuronidation. NS5A and NS5B inhibitors all are substrates or inhibitors of BCRP and p-glycoproteins. Concomitant drugs that compete for the same transporters may result in increased toxicity or reduced therapeutic effect. Recently, bradycardia and asystole associated with the use of sofosbuvir with another DAA and amiodarone highlighted the need to assess drug interactions from a drug transporter perspective [223]. Until this gap in knowledge is filled, it is paramount to report unexpected adverse reactions with the use of DAAs. Methadone has the potential to interact with DAAs as it is metabolized by CYP2C19 and 3A4. Buprenorphine and naloxone are metabolized through CYP3A4 and UGT1A1/3. Simeprevir and paritaprevir with ritonavir (in combination with ombitasvir and dasabuvir) have been shown to be safe in patients on opioid replacement therapy [224, 225]. Consistent with its metabolic profile, paritaprevir/ritonavir/ombitasvir and dasabuvir increased buprenorphine and naloxone exposure 107% and 28%, which required no dosage adjustment [226].

The management of these complex medication combinations requires expert knowledge. Substitution or safe discontinuation of the interacting drug can be attempted after careful evaluation of the benefit-risk ratio.

*Recommendations*

(38) Assessment and monitoring of drug-drug interactions between direct acting agents and commonly prescribed medications should occur at baseline and at frequent intervals during HCV therapy (Class 1, Level C).
(39) Ensuring that medication records are up to date, use of a systematic approach to identify combinations of potential concern, consulting pertinent HIV and/or HCV drug interaction resources (e.g., http://www.hiv-druginteractions.org/, http://www.hivclinic.ca/, http://www.hep-druginteractions.org/, and http://www.hcvdruginfo.ca/), and frequent patient monitoring are recommended to mitigate drug-drug interaction risk (Class 1, Level C).
(40) Nonessential medications should be discontinued for the duration of HCV treatment, particularly when HCV DAAs are used (Class 1, Level C).

## 8. Therapy in Special Populations 

### 8.1. Therapy for Acute HCV Infection in HIV-Infected Patients

The recognition that sexual transmission is a risk factor for acute HCV-infected in HIV-infected MSM populations has increased the need for periodic screening and consideration of rapid initiation of HCV antiviral treatment [235–237]. Small studies have evaluated the use of nonribavirin containing regimens and noted lower SVR rates with this approach [238, 239]. As a consequence, the European NEAT consensus panel on the management of acute HCV currently recommends standard doses of pegylated interferon and weight-based ribavirin therapy for treatment of acute HCV [50]. In cases where a rapid virologic response (RVR, defined as HCV RNA undetectable at week 4 of therapy) is achieved, 24 weeks of therapy are recommended, with full 48-week therapy for those without RVR [50]. Outcomes for those treated within 12–24 weeks of acquisition of HCV are higher than if therapy is delayed for over one year [238, 239].

Historically, acute HCV infection was managed with interferon-based antiviral treatment [50]. Multiple studies demonstrated improved SVR rates with interferon-based treatment of acute infection compared to cure rates achieved in chronic infection [238, 239]. The emergence of DAA-based therapy provides reason to reassess this paradigm. The high SVR rates achieved with DAA regimens reduce the urgency to initiate therapy within the six-month window following infection. In fact, waiting at least six months after infection will ensure that no patient destined to spontaneously clear acute infection will receive antiviral medications unnecessary. Although there are minimal data demonstrating interferon-free DAA therapy efficacy in the context of acute infection, there is no reason to believe that it will be less than observed in chronic infection. Optimal content and duration of DAA-based therapy in acute infection remains unstudied. On the other hand, treating immediately will diminish the risk for loss-to-follow-up if HCV antiviral treatment is deferred. It may also reduce the risk of further HCV transmission within the community in which the patient is participating in high risk activities. A concerted effort to provide education and resources to reduce reinfection risk behaviours is recommended.

*Recommendations*

(41) Patients participating in high risk activities for HCV infection or presenting with signs and symptoms of acute infection should be screened for HCV (Grade I, level C).

### 8.2. Pregnancy

In cohorts of greater than 3000 pregnant patients, HCV seroprevalence has been found to range from 0.1% to 2.4% [240]. There is no clinically significant impact of pregnancy on the characteristics of HCV or disease progression. Acute HCV infection during pregnancy is an uncommon event. However, there are rare reports of fulminant hepatitis resulting from acute infection in the context of pregnancy. There are no negative impacts of HCV on pregnancy-related outcomes. Specifically, no incremental rates of spontaneous abortion, prematurity, or obstetrical complications have been reported [241–243].

HCV vertical transmission risk has been reported to be approximately 1.7% in HCV seropositive women and 4.3% in HCV viremic women. The risk of vertical transmission is much higher in HIV-HCV coinfection (estimated to be as high as 19.4%) [244]. Other risk factors for HCV vertical transmission include high HCV viral load and instrumentation during delivery.

Although HCV virus is detected in breast milk, the risk of transmission with breast feeding is low unless the nursing mother has cracked or bleeding nipples. Breast feeding is not contraindicated in women with HCV. However, breast feeding is not routinely recommended in HIV-HCV coinfection due to the potential risk of HIV transmission.

HCV treatment options for pregnant patients are limited. Pegylated interferon is poorly tolerated in pregnancy and ribavirin is contraindicated due to teratogenicity. It is recommended that ribavirin be avoided for 6 months prior to conception in both females and their male partners. Women of childbearing potential and nonvasectomized HCV-infected men with female partners of childbearing potential are advised to utilize two forms of contraception while ribavirin is used. DAA safety and efficacy data in pregnancy are lacking.

*Recommendations*

(42) Pegylated interferon and ribavirin are contraindicated during pregnancy and 6 months prior to conception (Class 1, Level C).
(43) Women of childbearing potential and nonvasectomized HCV positive men with female partners of childbearing potential on ribavirin therapy should use 2 forms of contraception during treatment and for 6 months after treatment (Class 1, Level C).
(44) HCV positive women can safely breast feed. In HIV-HCV coinfection, breast feeding is contraindicated given HIV transmission risk (Class 2A, Level C).
(45) HCV positive pregnant women should not be offered HCV DAA therapy at present given the absence of safety and efficacy data and the fact that short-term deferral of therapy is rarely harmful (Class 2A, Level C).

### 8.3. Pediatric Population

Globally there are an estimated 11 million viremic pediatric cases of HCV infection (defined as <15 years of age) [245]. The relative child-to-adult ratio of HCV prevalence is 54% in low income countries, 28% in lower middle income countries, 21% in upper middle income countries, and only 4% in high income countries. There are differences in the natural history of pediatric HCV infection compared to adults. For example, there is a lower rate of progression to chronic HCV following acute infection (70% in adults versus 50–60% in pediatrics) [246]. Of the 5–10% of children who are infected HCV at birth, 25–75% will spontaneously clear the virus by ages 2-3. In contrast, children who acquire the infection as adolescents or in late childhood have the same natural history of HCV progression as adults. Studies following vertically exposed children over 10–20 years suggest that only 5–10% progress to advanced fibrosis and less than 5% are cirrhotic by adulthood [247]. Clinically, HCV-infected children are asymptomatic and have normal range to minimally elevated liver enzymes. Unique diagnostic considerations exist for HCV testing in infants given the potential for maternal transfer of HCV antibody which complicate interpretation of this measure. Positive HCV antibody test should be confirmed by HCV RNA testing. Chronic HCV infection is defined by evidence of continued viremia at age 3 or older. Treatment decisions of HCV in the pediatric population should consider similar factors as in adulthood. Treatment with pegylated interferon and ribavirin has demonstrated comparable SVR rates of 53% to adults [248–251]. Although it is anticipated that DAA treatment outcomes will be similar to adults there are currently no safety and efficacy data in the pediatric population.

*Recommendations*

(46) Screening for HIV and HCV is recommended for children with potential risk factors for exposure to these viruses (e.g., born to parents with HIV and/or HCV).
(47) All HIV-HCV coinfected infants should have HCV viremia testing done at ages 1, 2, and 3 given the high rate of spontaneous clearance before the age of 3 (Class 1, Level B).
(48) Current standard of treatment for HCV in pediatrics is weight-based pegylated interferon and ribavirin for those with advanced fibrosis. Consultation with a HCV pediatric specialist is recommended for patients with cirrhosis (Class 1, Level B).
(49) Children with minimal fibrosis should delay treatment for approval of interferon-free DAA regimens (Class 1, Level C).

## 9. Adverse Events and Adherence Management 

### 9.1. Adverse Events and Management

Historically, treatment regimens for HCV containing pegylated interferon and ribavirin had numerous side effects, many of which overlapped with side effects from HIV antiretrovirals. An extensive review of the side effects of interferon-based therapy and their management is beyond the scope of this paper but can be found elsewhere [252]. In the new paradigm of interferon-free therapy for HCV, adverse event profiles have improved dramatically. Interferon-free regimens do not appear to have a significant increased incidence of adverse events when used in coinfected individuals compared to monoinfected individuals, but drug-drug interactions are complex and patients must be monitored closely and in an expert setting.

Ledipasvir-sofosbuvir was well-tolerated in a large open-label phase III study involving 335 coinfected patients (ION-4) treated for HCV genotype 1 [166]. No patients discontinued treatment due to adverse events, serious adverse events only occurred in 2% of patients, and a single death occurred in a patient who developed endocarditis and sepsis secondary to injection drug use. The majority of adverse events were grade 1 or 2, with the most common adverse events being headache, fatigue, and diarrhea. The overall adverse event profile for ledipasvir-sofosbuvir in coinfected individuals is comparable to that of monoinfected individuals, although, in the ION-3 study of HCV monoinfected individuals, more patients developed fatigue and nausea [164].

Ledipasvir-sofosbuvir when coadministrated with tenofovir disoproxil fumarate (TDF) causes an increase in tenofovir levels, but the clinical significance of these increased levels remains unclear. In ION-4, 4 patients (1%) developed an increase in serum creatinine greater than 35 *μ*mol/L (0.4 mg/dL). Two of these patients completed their treatment for hepatitis C without any change in their antiretroviral regimen, one patient was switched from TDF to a different NRTI agent, and one patient had their dosage of TDF reduced. It is recommended that patients receiving a combination of TDF in combination with ledipasvir-sofosbuvir should be monitored closely for renal toxicity.

Ombitasvir-paritaprevir-ritonavir plus dasabuvir with ribavirin was studied in coinfected individuals in an open-label study that included 63 patients (TURQUOISE-1) [174]. No patients discontinued therapy due to an adverse event, although treatment-related adverse events were high (89%). The majority of these were mild or moderate, and only two severe adverse events were reported (insomnia and a tooth abscess). Anemia was uncommon; 6 patients required a reduction in ribavirin dosing because of anemia, and no patients experienced a grade 3 decline in hemoglobin (<80 g/L). The most common adverse events included fatigue, insomnia, nausea, and headache. Rises in total bilirubin were common.

Treatment with sofosbuvir combined with weight-based ribavirin was examined in treatment-naïve and treatment-experienced individuals with HIV and HCV coinfection in the PHOTON-1 and PHOTON-2 studies [187, 188]. Adverse events were common but serious adverse events were uncommon, and only a small number of patients discontinued therapy due to an adverse event. Decreases in hemoglobin and hyperbilirubinemia were common, and 11–19% of patients required a dose reduction of ribavirin. Decreases in CD4 count from baseline to end of treatment were common but recovered after treatment. Six patients experienced transient HIV virologic breakthrough, but none required a change in their antiretroviral regimen.

Ribavirin is well-described to cause predictable effects in HIV-positive individuals, including lymphopenia and transient hyperbilirubinemia secondary to ribavirin-induced hemolysis, particularly in patients receiving atazanavir [253, 254]. Anemia secondary to ribavirin remains common, but in PHOTON-1 and PHOTON-2, patients with HCV genotypes 2 and 3 experienced lower rates of anemia compared to those historically treated with interferon and ribavirin, due to the absence of bone marrow suppression secondary to interferon [187, 188]. Strategies to manage anemia in patients receiving a ribavirin containing regimen include dose reduction of ribavirin, the use of erythropoietin, and transfusion. The most common strategy clinicians utilize to manage anemia is dose reduction of ribavirin. While overall experience with coinfected patients remains low, dose reduction of ribavirin in interferon-free regimens does not appear to decrease rates of SVR. There are few published data on the use of erythropoietin in coinfected individuals.

ALLY-2 investigated the combination of daclatasvir and sofosbuvir in treatment-naïve and treatment-experienced patients with HCV genotypes 1–4 coinfected with HIV [180]. No patients discontinued therapy due to adverse events, and a single death reported in the study was due to cardiac arrest in a patient with multiple comorbidities and not deemed secondary to therapy. Grade 3 or 4 lab abnormalities were rare. Two patients experienced virologic breakthrough with HIV RNA ≥400 copies/mL at the end of therapy. In both of these patients, no resistance mutations were detected and one patient resuppressed without any change in antiretroviral therapy while the second patient was lost to follow-up due to incarceration.

There are no dedication sofosbuvir-simeprevir studies in HIV-HCV coinfection to provide insights into side effect profile. In the COSMOS study of HCV monoinfected participants, common side effects included fatigue (25%), headache (21%), nausea (17%), insomnia (14%), and pruritus (11%). Other noteworthy side effects with sofosbuvir-simeprevir for 12 weeks included rash (11%) and photosensitivity reactions (7%) [230].

*Recommendations*

(50) Close monitoring for side effects during HCV therapy is required (Class 1, Level C).
(51) Anemia related to HCV treatment either with pegylated interferon/ribavirin or a DAA with ribavirin should be primarily managed with ribavirin dose reduction. Erythropoietin use is not recommended for first line anemia management (Class 2b, Level B).

## 10. HIV and Liver Transplantation

The management of end-stage liver disease includes orthotopic liver transplantation. Guidelines for liver transplant have been developed in both Europe and the United States [255–257]. In addition to meeting requirements for liver transplantation, HIV-infected patients must demonstrate virologic suppression and CD4 counts >200 cells/*μ*L and must have no history of recent opportunistic infections. Outcomes of transplant amongst HIV-infected individuals have been evaluated in several European and American cohorts. Overall, short-term outcomes are comparable to the general transplant populations, while the hazard ratio for long-term survival is reduced by approximately 2-fold [255, 258–260]. Prior reviews suggest that the 5-year survival post-liver transplantation in HIV-HCV coinfection is in the 50–55% range [258, 261, 262]. A previous review of the transplant program in Spain found a five-year survival rate of 54% in HIV-HCV coinfected recipients (*n* = 84) compared to 71% for HCV monoinfected patients [262]. These reduced survival rates in coinfected patients are driven primarily by HCV-induced liver disease. Diminished survival outcomes have raised questions regarding liver transplantation in the HIV-HCV coinfection context. Of note, the single most important predictor of outcome is posttransplant HCV viral elimination with HCV antiviral treatment. In those achieving a SVR the 5-year survival is approximately 80% [255]. The promise of DAA-based therapeutic success in posttransplant HIV-HCV coinfected patients raises hopes that the gap in survival will be narrowed.

HCV antiviral treatment following transplant is complex but far more feasible with interferon-free DAA regimens [263]. DAA therapy in the posttransplant context has been assessed in HCV monoinfection but not HIV-HCV coinfection. Safety, tolerability, and efficacy have been established in HCV monoinfection with several regimens including sofosbuvir-ribavirin [264], simeprevir-sofosbuvir [176], and sofosbuvir-ledipasvir [265].

Liver transplant patients on immunosuppressive drugs are at particular risk for serious drug interactions since cyclosporine and tacrolimus metabolism are highly dependent on CYP3A4 [266]. DAA interaction with cyclosporine in healthy volunteers led to an increase of cyclosporine exposure by 2.7- and 4.6-fold, respectively [255, 263, 267–269]. Cyclosporine was also found to increase simeprevir exposure by 4.8-fold. As a result, simeprevir is not recommended for coadministration with cyclosporine [230]. The magnitude of the interaction was greater with tacrolimus, with an increase by 17- and 70-fold for protease inhibitor exposure. The decision to coadminister current DAAs with cyclosporine or tacrolimus should be made on a case by case basis with the support of experts in pharmacology, hepatology, and infectious diseases [270, 271].

*Recommendation*

(52) HIV-HCV coinfected patients should be considered for liver transplantation assuming all necessary criteria are met (Class 2a, Level C).
(53) HCV antiviral therapy should be considered in post-liver transplant recipients (Class 1, Level C).

## 11. Timing of Initiation of HCV Therapy in the Era of DAAs

Access to standard of care antiviral therapy when clinically indicated has long been recommended in Canada by experts involved in the care of patients living with HCV [7] and we continue to advocate for this for HIV-HCV coinfected patients. The authors recognize that due to restrictions to access and reimbursement of HCV antiviral drugs regimens for HCV, clinicians and patients may face difficult decisions regarding therapy. In this situation alternate options may be considered including deferral of therapy. Individuals with minimal fibrosis may be able to defer therapy compared to those with more advanced disease, as they have lower risk of medium-term progression of disease. These individuals may be able to wait for future combinations and potentially improved access to HCV antiviral therapy. If deferral of therapy is necessary, updated staging for fibrosis progression is recommended on an annual basis if access to transient elastography is possible, or every 3 years if liver biopsy is to be performed.

Additional considerations of patient readiness and consideration of possible onward HCV transmission risk for individuals in a core transmitter group (IDU and certain MSM populations) compared to those without high risk for transmission (e.g., many baby boomers (born approximately 1945–1970)) may influence a decision to consider delaying therapy.

Circumstances may exist in which first line regimens are not accessible to patients (e.g., restricted funding). The above second line regimens could be considered as treatment options. However, the patient must be fully aware of the diminished likelihood for cure and/or increased likelihood for adverse events compared to first line regimens. Furthermore, lack of provincial availability of some DAAs may preclude use.

## 12. Conclusions

HIV-HCV coinfection is common in Canada and associated with a heavy burden of concurrent comorbid conditions which affect health status and outcomes. As such, harm reduction strategies should be implemented to decrease risk of infection amongst high risk populations such as injection drug users and incarcerated individuals.

Coinfection is associated with increased risk of progression of liver disease. End-stage liver disease is a chief cause of morbidity and mortality amongst coinfected individuals.

All HIV-HCV coinfected individuals should be assessed for HCV therapy. ART initiation, irrespective of CD4 count, is an effective strategy to slow liver disease progression and is consistent with current HIV treatment guideline recommendations. However, HCV antiviral treatment initiation prior to HIV ARV therapy in patients with high CD4 cell counts (>500 cells/*μ*L) avoids drug-drug interactions, diminishes pill burden issues due to concomitant HIV and HCV medication dosing and may improve future tolerability of ARV [4].

DAA treatment has revolutionized HCV treatment in the HIV-HCV coinfected population providing highly effective, short duration, well-tolerated, and safe treatment options. In fact, SVR rates achieved in HIV-HCV coinfection are similar to HCV monoinfection. Current standard of care for genotype 1-infected patients consists of interferon-free, combination DAA regimens. Careful assessment of drug-drug interactions with ART and other common medications is necessary when using these agents.

Current standard of care for genotypes 2 and 3 infected patients remains dual therapy with sofosbuvir and ribavirin as well as daclatasvir with sofosbuvir. When included in DAA regimens, weight-based dosing of ribavirin is recommended. In individuals with mild liver disease, conservative monitoring with deferral of therapy may be necessary given current HCV DAA funding restrictions. Due to current reimbursement restrictions in some jurisdictions, pegylated interferon and ribavirin may represent the only treatment option available for nongenotype 1 infection. This is not acceptable and should be changed immediately to allow for the provision of optimal patient care.

## Figures and Tables

**Table 1:** Grading system for recommendations.

Classification description	
Class of evidence	
Class 1	Conditions for which there is evidence and/or general agreement that a given diagnostic evaluation procedure or treatment is beneficial, useful, and effective
Class 2	Conditions for which there is conflicting evidence and/or a divergence of opinion about the usefulness/efficacy of a diagnostic evaluation, procedure, or treatment
Class 2a	Weight of evidence/opinion is in favour of usefulness/efficacy
Class 2b	Usefulness/efficacy is less well established by evidence/opinion
Class 3	Conditions for which there is evidence and/or general agreement that a diagnostic evaluation and procedure/treatment is not useful/effective and in some cases may be harmful

Grade of evidence	
Level A	Data derived from multiple randomized clinical trials or meta-analyses
Level B	Data derived from a single randomized trial or nonrandomized studies
Level C	Only consensus opinions of experts, case studies, or standard-of-care

Adapted from [7].

**Table 2:** Baseline assessment of coinfected patients.

	Test	Comment
Viral hepatitis screens	HCV antibody	
	Quantitative HCV RNA	
	HCV genotype	
	Hepatitis B surface antigen	Chronic HBV infection
	Hepatitis B surface antibody	Immunity to HBV
	Hepatitis B core antibody	
	Hepatitis A IgG	If negative, indicates need for HAV vaccine

Liver-related	Complete blood count	Thrombocytopenia may indicate advanced liver disease
	ALT, AST	
	ALP, GGT	
	Albumin, INR, and total bilirubin	Abnormalities suggest advanced liver disease
	Ultrasound	
	Fibroscan	

Screens for other chronic conditions of liver disease	Alpha-1-antitrypsin	Alpha-1 antitrypsin deficiency
	Antinuclear antibody, anti-smooth muscle antibody	Autoimmune hepatitis
	Anti-mitochondrial antibody	Primary biliary cholangitis
	Ceruloplasmin	Wilson's disease
	Iron saturation	Hemochromatosis
	Lipid Profile	Fatty liver disease
	TSH	Autoimmune thyroiditis
	Immunoglobulins A, G, and M	Autoimmune hepatitis, primary biliary cholangitis, and alcoholic liver disease

**Table 3:** Criteria for interpretation of transient elastography in HIV-HCV coinfected patients [115, 116].

Score (kilo Pascals, kPa)	Metavir equivalent	Interpretation
≤7.2	F0/1	Mild fibrosis
7.2–9.5	F2	Moderate fibrosis
9.5–12.5	F3	Advanced fibrosis
>12.5	F4	Severe fibrosis/cirrhosis

**Table 4:** Drug-drug interactions between antiretroviral agents and directly acting antivirals for hepatitis C.

Usual doses	90 mg/400 mg daily	150/100/25 mg daily + 250 mg BID with food	150 mg daily and 400 mg daily with food	60 mg daily plus 400 mg daily

Nucleoside/nucleotide reverse transcriptase inhibitors				
Abacavir/lamivudine	18% ↑ AUC, 10%, ↑ *C*_max_ and 26% ↑ *C*_min_ of ledipasvir. 21% ↑ AUC of sofosbuvir, not considered clinically significant. No dose adjustment required (Harvoni PM),^§^	Coadministration has not been studied but no clinically significant drug interaction expected.^§^	Coadministration has not been studied but no clinically significant drug interaction is expected.^§^	Coadministration has not been studied but no clinically significant drug interaction is expected.^§^
Tenofovir disoproxil fumarate (TDF)/emtricitabine	TDF exposures are increased (AUC 40–98%, *C*_max_ 32–79% and *C*_min_ 47–163%) when ledipasvir/sofosbuvir is coadministered with TDF-containing antiretroviral regimens, including NNRTIs, boosted PIs, and integrase inhibitors. Appropriate monitoring for TDF-associated toxicity is recommended (Harvoni PM).^‡^	No clinically significant changes. No dose adjustment required (Holkira Pak PM)^§^	No clinically significant changes in pharmacokinetics of TDF, simeprevir, or sofosbuvir noted. No dose adjustment is required (Galexos PM, Harvoni PM).^§^	No clinically significant changes in pharmacokinetics of TDF, daclatasvir, or sofosbuvir noted. No dose adjustment is required (Galexos PM, Harvoni PM).^§^

Integrase strand transfer inhibitors				
Dolutegravir	TDF exposures were 65–115% higher when ledipasvir/sofosbuvir was coadministered with dolutegravir plus TDF DF/emtricitabine. Ledipasvir/sofosbuvir may be coadministered with dolutegravir. If TDF DF/emtricitabine is included as an NRTI backbone, appropriate monitoring for TDF-associated toxicities is recommended [227].^‡^	Dolutegravir exposures increased 22–38% while paritaprevir *C*_trough_ ↓ 34%. These changes are not considered clinically significant and dolutegravir may be administered with the 3D regimen without dose adjustment [228].^§^	Coadministration has not been studied but no clinically significant drug interaction is expected.^§^	No clinically significant changes in pharmacokinetics of dolutegravir or daclatasvir noted. No dose adjustment is required [229].^§^
Elvitegravir/cobicistat	Increased TDF exposures anticipated with coadministration; appropriate monitoring for TDF-associated toxicities is recommended [227] (Harvoni PM). Increased cobicistat exposure. Clinical significance unknown but likely not clinically relevant [227].^‡^	Coadministration has not been studied but cobicistat is expected to increase paritaprevir and ritonavir concentrations (Holkira Pak PM). Coadministration *cannot be recommended*.^†^	*Not recommended* with cobicistat-boosted regimens due to risk of significantly increased simeprevir concentrations [230, 231].^†^	Potential for increased daclatasvir exposures due to CYP3A4 inhibition by cobicistat. Reduce daclatasvir dose to 30 mg once daily when coadministering with cobicistat-based regimens (Daklinza PM).^§^
Raltegravir	No clinically significant changes noted with coadministration. No dose adjustment required (Harvoni PM).^§^	No clinically significant changes noted with coadministration. No dose adjustment required (Holkira Pak PM).^§^	No clinically significant changes noted with coadministration. No dose adjustment is required [230, 232, 233].^§^	Coadministration has not been studied but no clinically significant drug interaction is expected (Daklinza PM).^§^

Nonnucleoside reverse transcriptase inhibitors				
Efavirenz	In combination with TDF/FTC, no clinically significant changes in sofosbuvir or efavirenz pharmacokinetics were noted, while tenofovir AUC ↑ 98% and *C*_min_ ↑ 163%. Appropriate monitoring for tenofovir-associated toxicities is recommended [227] when the combination of efavirenz, tenofovir DF, and FTC is coadministered with ledipasvir/sofosbuvir (Harvoni PM).^‡^	Coadministration of efavirenz based regimens with paritaprevir, ritonavir plus dasabuvir is *contraindicated* due to poor tolerance and liver enzyme elevations (Holkira Pak PM).^†^	91% ↓ *C*_min_, 71% ↓ AUC of simeprevir. *Avoid combination* [230, 231].^†^	Daclatasvir exposures are decreased with coadministration. Increase daclatasvir to 90 mg once daily with efavirenz (Daklinza PM).^‡^
Etravirine	Coadministration has not been studied.^‡^	*Contraindicated* with etravirine due to risk of decreased paritaprevir, ombitasvir, and dasabuvir concentrations (Holkira Pak PM).^†^	*Not recommended* with etravirine due to risk of decreased simeprevir concentrations [230].^†^	Coadministration has not been studied. Potential for decreased daclatasvir concentrations; *avoid coadministration* until further data available.^†^
Rilpivirine	In combination with TDF/FTC, no clinically significant changes in sofosbuvir or rilpivirine pharmacokinetics were noted, while tenofovir AUC ↑ 40% and *C*_min_ ↑ 91%. Appropriate monitoring for tenofovir-associated toxicities is recommended [227] when the combination of rilpivirine, tenofovir, and FTC is coadministered with ledipasvir/sofosbuvir (Harvoni PM).^‡^	3.25-fold ↑ AUC, 2.55-fold ↑ *C*_max_, and 3.62-fold ↑ *C*_min_ of rilpivirine, not mitigated by staggered administration. Coadministration is *not recommended* due to increased risk for prolonged QTc (Holkira Pak PM).^†^	No clinically significant changes noted with coadministration. No dose adjustment required [233].^§^	Coadministration has not been studied but no clinically significant drug interaction expected (Daklinza PM).^§^

Protease inhibitors				
Atazanavir/ritonavir	75% ↑ *C*_min_, 33% ↑ AUC of atazanavir. 2.13-fold ↑ AUC, 1.98-fold ↑ *C*_max_, and 2.36-fold ↑ *C*_min_ of ledipasvir. No dose adjustment required (Harvoni PM). Monitor for atazanavir toxicity (e.g., hyperbilirubinemia).^§^	Atazanavir should be taken without additional ritonavir with the 3D regimen (Holkira Pak PM).^‡^	*Not recommended* with ritonavir, boosted or unboosted HIV protease inhibitors due to risk of significantly increased simeprevir concentrations [230].^†^	Reduce dose of daclatasvir to 30 mg once daily when coadministering with atazanavir/ritonavir (Daklinza PM).^‡^
Atazanavir/cobicistat	Combination is not studied. In combination with elvitegravir/cobicistat, cobicistat exposure is increased. Clinical significance is unknown but likely not clinically relevant [227]. Monitor for atazanavir toxicity (e.g., hyperbilirubinemia).^§^	Atazanavir plus cobicistat is *not recommended* with HOLKIRA*™* PAK (Holkira Pak PM).^†^	*Not recommended* with cobicistat due to risk of significantly increased simeprevir concentrations [230].^†^	Reduce dose of daclatasvir to 30 mg once daily when coadministering with cobicistat (Daklinza PM).^‡^
Darunavir/ritonavir	No changes in darunavir pharmacokinetic parameters; 39% ↑ AUC, 45% ↑ *C*_max_, and ↑ 39% *C*_min_ of ledipasvir. Changes not considered clinically significant. No dose adjustment is required (Harvoni PM).^§^	24% ↓ AUC, 8% ↓ *C*_max_, and 48% ↓ *C*_min_ of darunavir 800 mg daily. Darunavir should be taken without additional ritonavir with the 3D regimen since ritonavir is already included. Monitor for HIV viral breakthrough (Holkira PM).^‡^	2.59-fold ↑ AUC, 1.79-fold ↑ *C*_max_, and 4.58-fold ↑ *C*_min_ of simeprevir and 18% ↑ AUC, 31% ↑ *C*_min_ of darunavir. Coadministration *not recommended* [230].^†^	Daclatasvir AUC increased 41%, *C*_max_ decreased 23% with coadministration. Changes not considered clinically significant. No dose adjustment is required [234].^§^
Darunavir/cobicistat	Coadministration has not been studied but no clinically significant drug interaction expected. In combination with elvitegravir/cobicistat, cobicistat exposure is increased. Clinical significance is unknown but likely not clinically relevant [227].^§^	Darunavir plus cobicistat is *not recommended* with the 3D regimen, which already includes ritonavir (Holkira PM).^†^	*Not recommended* with cobicistat due to risk of significantly increased simeprevir concentrations [230].^†^	Coadministration has not been studied but no clinically significant drug interaction is expected.^§^
Lopinavir/ritonavir	Coadministration has not been studied. Significant drug interaction not anticipated.^§^	*Should not be coadministered* with HOLKIRA PAK due to the potential for an increase in paritaprevir exposures (Holkira Pak PM).^†^	*Not recommended* with ritonavir, boosted or unboosted HIV protease inhibitors due to risk of significantly increased simeprevir concentrations [230].^†^	No clinically significant changes noted with coadministration. No dose adjustment required [234].^§^

CCR5 antagonist				
Maraviroc	Coadministration has not been studied but no clinically significant drug interaction expected.^§^	Coadministration has not been studied but maraviroc exposure is expected to be increased by ritonavir. Reduce maraviroc to 150 mg BID or 300 mg daily.^‡^	Coadministration has not been studied but no clinically significant drug interaction is expected (Galexos PM).^§^	Coadministration has not been studied but no clinically significant drug interaction is expected (Daklinza PM).^§^

Key: ^†^avoid combination; ^‡^caution/dose adjustment; ^§^acceptable combination OK.

AUC: area under the curve; *C*_min_: concentration minimum; *C*_max_: peak concentration; *C*_trough_: trough concentration; BID: twice a day; NNRTI: nonnucleoside reverse transcriptase inhibitor; PI: protease inhibitor; PM: product monogram.

**Table 5:** Summary of antiretroviral regimen recommendations for patients who require concomitant HIV and hepatitis C treatment.

	Recommended	Alternative	Not recommended
Sofosbuvir 400 mg/ledipasvir 90 mg once daily	No restrictions with first or second line ART regimens		In patients with preexisting renal dysfunction or significant risk factors for nephrotoxicity: may wish to avoid tenofovir-containing regimens due to potential for ↑ tenofovir concentrations

Paritaprevir 150 mg/ritonavir 100 mg/ombitasvir 25 mg once daily + dasabuvir 250 mg BID with food	Atazanavir (without additional ritonavir), raltegravir, and Dolutegravir	Darunavir (without additional ritonavir)	Ritonavir- or cobicistat-boosted regimens; efavirenz, etravirine, and rilpivirine

Simeprevir 150 mg daily plus sofosbuvir 400 mg daily with food	Dolutegravir, raltegravir, or rilpivirine-based regimens		Ritonavir- or cobicistat-boosted regimens; efavirenz, etravirine, and nevirapine

Daclatasvir 60 mg daily plus sofosbuvir 400 mg daily	Atazanavir (requires decrease in daclatasvir dose to 30 mg daily), darunavir, dolutegravir, raltegravir, or rilpivirine-based regimens	Efavirenz (requires increase in daclatasvir dose to 90 mg daily)	Etravirine and nevirapine

BID: twice daily.
